# Monitoring Distracted Driving Behaviours with Smartphones: An Extended Systematic Literature Review

**DOI:** 10.3390/s23177505

**Published:** 2023-08-29

**Authors:** Efi Papatheocharous, Christian Kaiser, Johanna Moser, Alexander Stocker

**Affiliations:** 1 RISE Research Institutes of Sweden, SE-164 40 Kista, Sweden; 2 Virtual Vehicle Research GmbH, 8010 Graz, Austria; christian.kaiser@v2c2.at (C.K.); johanna.moser@v2c2.at (J.M.); alexander.stocker@v2c2.at (A.S.); 3 KTM AG, 5230 Mattighofen, Austria

**Keywords:** smartphone, smartphone sensors, driver monitoring, driver distraction detection

## Abstract

Driver behaviour monitoring is a broad area of research, with a variety of methods and approaches. Distraction from the use of electronic devices, such as smartphones for texting or talking on the phone, is one of the leading causes of vehicle accidents. With the increasing number of sensors available in vehicles, there is an abundance of data available to monitor driver behaviour, but it has only been available to vehicle manufacturers and, to a limited extent, through proprietary solutions. Recently, research and practice have shifted the paradigm to the use of smartphones for driver monitoring and have fuelled efforts to support driving safety. This systematic review paper extends a preliminary, previously carried out author-centric literature review on smartphone-based driver monitoring approaches using snowballing search methods to illustrate the opportunities in using smartphones for driver distraction detection. Specifically, the paper reviews smartphone-based approaches to distracted driving behaviour detection, the smartphone sensors and detection methods applied, and the results obtained.

## 1. Introduction and Motivation

Driving a vehicle is a cognitively demanding task, and driver distraction and inattention, in general, have been major concerns for many years, as both significantly increase the risk of accidents, especially for younger drivers (cf. e.g., [1,2,3,4]).

Driver distraction is the “diversion of attention away from safe driving activities to a competing activity” [5,6] and occurs when “a driver is delayed in recognising information needed to safely perform the driving task, because an event, activity, object, or person within or outside the vehicle forces or induces the driver to shift attention away from the driving task” [7]. Drivers may increasingly shift their attention from the driving task to non-driving-related secondary tasks, for example, by taking their hands (manual distraction), eyes (visual distraction), and/or mind (cognitive distraction) away from driving (cf. e.g., [8,9]).

Driver distraction by secondary tasks, i.e., smartphone use, is one of the main causes of road accidents (cf. e.g., [10,11,12]), while avoiding road accidents has always been a driving force for technological progress. Consequently, the detection of driver distraction has become a popular research topic (cf. e.g., [13,14,15]), and vehicle manufacturers will increasingly implement proprietary distraction detection systems to prevent accidents (cf. e.g., [16,17]). As modern vehicles have become computers on wheels equipped with a plethora of sensors [18,19], distraction detection systems can integrate the data generated by vehicles during operation and infer certain types of distraction. However, distraction detection systems can also be based on additional hardware and software that is brought into the vehicle, such as smartphones [20,21,22]. Modern smartphones have a variety of embedded sensors that can track position, rotation, and acceleration, as well as record audio and video (of the driver) that can be used to monitor distracted driving [22].

The aim of this paper is to extend a previously published preliminary literature review of scientific peer-reviewed published work on driver distraction using smartphones [23]. Even though there have been several other literature reviews published on the topic of driver distraction, in general (e.g., [2,5,6,24]), and driver distraction monitoring (e.g., [3]), none of them have exclusively focused on distracted driver monitoring with smartphone-based systems.

Our literature review is based on a previously published work that focused on a rather small number of 16 selected papers and performs an extended author-centric literature review, including, in total, 65 selected papers and combining the well-established method of a systematic literature review with snowballing. This review method has been used to shed light on previously published smartphone-driven distracted driver behaviour monitoring studies using smartphones and compile the state of the art on this subject. More specifically, this paper collects smartphone-based approaches to distraction detection, the smartphone sensors and distraction detection methods used, and the results obtained. In doing so, we contribute to the academic literature on driver state monitoring and summarise the state of the art in smartphone-based distracted driver monitoring studies.

This paper is structured as follows: Section 2 includes the background on smartphone-based driver distraction detection, Section 3 presents the research method used, an extended systematic literature review combined with snowballing. Section 4 presents the results, a description of the papers selected and a summary of each of the papers studied, focusing on the aim of this work, the approach chosen, the detection method chosen, and the smartphone sensors used, as well as some concrete results obtained. Section 5 provides a discussion of the results, and Section 6 concludes the paper.

## 2. Background

The digital transformation wave is challenging the mobility and transport sector like no other sector, creating a paradigm shift towards electric and automated vehicles enabled by various digital technologies such as the Internet of Things, robotics, big data analytics, and artificial intelligence [25,26,27].

Hence, modern vehicles have evolved into networked computers on wheels and are equipped with a plethora of sensors, actuators, and information and decision-making systems to ensure driving functionality and assist drivers in their driving tasks [18,19,28]. For several years, the automotive industry has been investing heavily in vehicle automation to make driving safer, more efficient, and more comfortable [29]. However, despite great progress and increasingly powerful assistance systems, full vehicle automation at higher driving speeds and in a variety of driving scenarios is still a long way off [30]. For this reason alone, the human driver will continue to play an important role in the vehicle as the executor of the driving task in manual driving mode or as the operator of vehicle automation systems in automated driving mode, which is more than what was assumed a few years ago in the course of the vehicle automation hype. Driver monitoring will, therefore, remain an important challenge, even in the context of partially automated driving. This includes the prediction of driving behaviour, which has been a major area of research in recent years [31].

Driving a vehicle is a cognitively demanding task, and driver distraction [32] and, more generally, driver inattention [33,34] have been a major problem for many years, as both significantly increase the risk of accidents, especially for young drivers [35,36]. Distraction and inattention are obviously major problems when driving manually, but even when vehicle automation systems are activated, the driver must stay in the loop. Driving a vehicle with activated SAE Level 2 assistance systems [30], the maximum level of automation in most of today’s production vehicles, requires the driver to constantly monitor the vehicle’s automation systems and keep their hands on the steering wheel. If the driver removes their hands from the steering wheel, the vehicle automation system will remind the driver to reposition their hands after a period of time. In addition, if the vehicle automation system reaches its system limits, the driver must immediately take over the driving task again [30]. In both cases, driver distraction due to over-trust in partially automated driving is a major safety risk that has led to fatal accidents [37].

There have already been literature reviews on driver distraction detection published (cf. e.g., [2,3,6,24,32]); thus, we want to highlight how we distinguish our work from other reviews (cf. Table 1). One key difference is that using a smartphone as a tool or data source for approaches to prevent driver distraction is mandatory for the papers included in this review. This is different to Young et al. [2] or Oviedo-Trespalacios, O. [24], who focused on the different aspects of distraction coming from the use of mobile phones inside a car. Dong et al. [3] also mentioned smartphone-based systems, but not in detail. Kashevnik et al. [32] also had no emphasis on smartphones and created a holistic framework from sensors to specific approaches for detecting driver distraction. Lee et al. [6] concentrated on the elaboration of the definition of driver distraction, in general. In contrast to the mentioned published literature reviews, this work is a systematical literature review that also gives a more detailed and reflected view, as it contains both a short descriptive summary for each reviewed paper as well as an aggregated summary including tables with the study objective, analysis method, sensors used, and obtained results.

Distracted driving through the use of electronic devices, especially texting or other types of mobile phone use while driving, is a major risk factor for vehicle collisions [35], as it results in a secondary task that pulls the driver’s eyes off the road, the driver’s mind off driving, and the driver’s hands off the steering wheel. The use of modern smartphones while driving has, therefore, become one of the main causes of vehicle accidents with injuries and fatalities [38,39]. Research has shown that young drivers, in particular, touch their smartphones an average of 1.71 times per minute while driving for a variety of reasons, including texting, browsing the Internet, listening to music, or watching videos [40].

There are several causes of road accidents, which can also occur in combination. For instance, younger drivers are more likely to be involved in crashes due to inexperience, poor driving ability, and risk-taking behaviours including excessive speed and drug and alcohol use, while older drivers are more likely to be involved due to visual, cognitive, and mobility problems [41]. Traffic volume, weather and lighting conditions, and specific road sections are other causes of road accidents discussed in the scientific literature [42]. However, according to police officers, the use of mobile phones while driving is under-recorded in traffic accident records [41], and phone use has been shown to significantly reduce driver safety, which is of particular concern for young drivers, who have less driving experience and tend to use phones more [43].

On the other hand, smartphones have also become an important platform for mobile applications, in general, and for the transport and mobility sector, in particular. These include, for example, app-based vehicle information systems [44] or fleet management apps, which, if used appropriately, can even lead to the prevention of risky driving behaviour [45,46]. In addition, smartphone sensors and cameras are already being used to enable driver monitoring systems, and researchers are exploring new ways to improve detection accuracy.

Research has been exploring the use of the smartphone and integrated smartphone sensors as a basis for the development of app-based driver monitoring systems [47,48]. Smartphone-based driver monitoring systems would have the added value of being able to retrofit older vehicles with innovative camera and sensor technology, as they do not rely on the vehicle’s sensors and actuators [49,50]. Researchers have already demonstrated good accuracy in detecting distracting tasks from recorded images [48,51]. In addition to focusing on the original distraction monitoring challenge, there are several sub-topics addressed by the research, including driver detection (based on smartphone location), gaze [52], or head pose detection [53], or the detection of different driver behaviours, such as driver inattention [49], drowsiness [54], or risky driving [46].

As smartphone penetration continues to grow rapidly, so does the number of messages received (via email, instant messaging, etc.). According to the Pew Internet Survey [55], the share of Americans who own a smartphone has grown to 81% in 2019. As smartphones are increasingly used while driving, smartphone-based distractions have become a major problem [47]. This has exacerbated the case of phone-based distraction, as smartphones offer more communication options than traditional cell phones.

As not only the prevalence but also the computational power of smartphones has increased significantly, and as modern smartphones also allow the use of machine learning approaches, the authors expect that a number of studies using smartphone driver distraction detection systems have been published in recent years. Both smartphone-based data analysis and data-driven context-aware systems on smartphones have gained increasing attention in recent years [56].

However, the use of smartphones as driver monitoring systems remains a double-edged sword: the use of smartphones in vehicles can lead to driver distraction and accidents, but their advanced architecture and powerful hardware and software can enable driver monitoring applications that detect driver inattention and distraction, alert the driver, bring the driver back into the loop, and, thus, support driving safety. The smartphone can become a relevant platform for the development of driver monitoring systems, also in the context of increasing vehicle automation. The aim of this article is to provide a systematic overview of the current state of research in the development of smartphone-based driver monitoring systems.

## 3. Method

**Figure 1:**
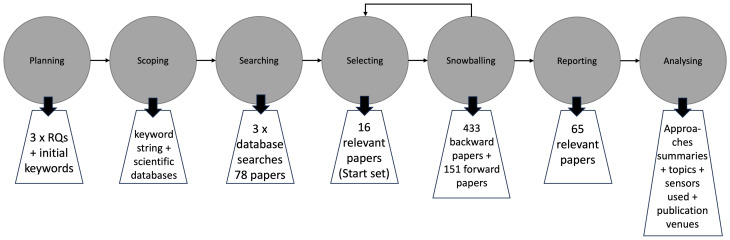
Methodology combining systematic literature review steps and snowballing search.

A systematic literature review [57,58,59] is the method chosen to study the topic, and it contains a set of steps: planning, scoping, searching, selecting, reporting, and analysing. A preliminary execution of the method has already been published [23], whereas as an extension to the method, a snowballing search complements the results reported and analysed in this work (cf. Figure 1).

### 3.1. Planning

At the planning step, the researchers defined the research questions (RQs) below:

- RQ1: What smartphone-based approaches for driver distraction detection have been published in the last ten years?
- RQ2: What smartphone sensors and detection methods have been used?
- RQ3: What tangible results have been achieved by using smartphone-based detection approaches?

Two researchers first developed the keyword string in three iterations, which was aimed to not be too specific and not too generic. After each iteration, both authors applied the string to several scientific databases and scanned the results. Based on the scan, they tried to further improve the keyword string to obtain the most promising search results.

### 3.2. Scoping

Once the keyword string was determined, the three scientific databases IEEE Xplore, Scopus, and Web of Science were selected (scoping phase). The databases were selected based on their popularity amongst scholars and academics, which is due to their focused and curated content, specialised coverage, reliable metadata, search tools, and citation analysis capabilities. The search results were imported into a Mendeley (Mendeley reference management software (https://www.mendeley.com/) (accessed on 24 July 2023)) group, and the metadata of the papers were completed when needed. The 78 papers were then imported into Rayyan (Software for Systematic Literature Reviews (https://rayyan.ai/) (accessed on 24 July 2023)) and blindly provided to the two researchers for analysis. In the selecting phase, the researchers each analysed all 78 papers, categorising them as “include”, “exclude”, and “maybe”, and adding notes and labels. Through three rounds of joint iteration, the researchers ultimately settled on 16 papers.

### 3.3. Searching

This step includes searching within a set of scientific databases as sources of information, namely, ACM, IEEE Xplore, Scopus, and Web of Science (WoS). The scientific databases and the search string were revised and agreed upon amongst the authors based on the quality of the results obtained. The keyword search string used across the databases is as follows: “(“*phone” AND (“sensor?” OR “data”) AND (“driver distraction” OR “driving distraction” OR “distracted driving”) AND (“detect*”) AND NOT (“simulat*”))”. The search was performed on 18 February 2021. The search string was used on the title, abstract, and keywords (hence, ACM digital library could not be used, as it does not provide this title, abstract, and keyword search). The researchers restricted the search to include papers from the last 10 years (2011–2021) due to the huge growth smartphones have had in the last decade and the changes in the automotive domain due to digitalisation. In Scopus, the search excluded all other subject areas (i.e., medicine) except engineering, computer science, and social sciences. In WoS, all the databases were used.

### 3.4. Selecting

Selecting papers from the scoping step (cf. Table 2) is a method to identify the most relevant publications to the RQs. The researchers carried out this step independently and with a blind process. After the blind process, the differences were discussed until an agreement was reached. As illustrated in Table 3, 78 papers (60 of them unique) were the input for the analysis, while the researchers agreed to use 20 of them (16 unique) for further analysis, using inclusion/exclusion criteria.

The following inclusion/exclusion criteria were agreed on:

1. Exclude results that are handbooks, Ph.D. theses, patents, or only abstracts;
2. Exclude results that are citations or conference proceedings;
3. Exclude duplicates and papers in languages other than English;
4. Exclude results that do not use smartphone data or phone data.

In particular, two researchers blindly looked at the 78 papers using the online tool Rayyan in an initial analysis phase. The results of the initial analysis of the two researchers were contrasted and discussed. At first glance, the results of the two researchers differed stronger than expected (see column “Initial analysis” in Table 4; in total, 19% conflicts). However, through two rounds of joint iteration, the researchers ultimately agreed and settled on 16 papers. In Iteration 1, the papers categorised as “maybe” were discussed jointly. Thereby, a clarification of detailed criteria for papers in the boundary was made (i.e., include papers utilising WiFi/Radio signal strengths from smartphones), as well as clarification on duplicates, where one researcher included the first entry, while the other researcher included the second entry of the duplicate. In the second iteration, the 15 remaining conflicts (where one researcher wanted to include it, while the other researcher wanted to exclude it) were resolved by jointly scanning and discussing the papers one more time.

However, in the subsequent phase (Iteration 3), when all papers were analysed in detail, the authors noticed that two papers did not use smartphone sensors at all, although at first glance, it looked like they did. In particular, Kim et al. [60] mentioned a “resource sharing device” and named the driver’s mobile phone as an example several times; however, they used Raspberry Pi to collect camera images as input for a driver monitoring system but stated that they planned to “verify the proposed system with a real device in the vehicle, such as the driver’s mobile phone” in the future. Saeed et al. [61] detected risky behaviour through differentiable patterns in received WiFi signals–patterns in a classification system, and drowsy and inattentive driving are classified into four main gestures that reflect unsafe driving. These gestures include (a) yawning, (b) head jerks, (c) sideways motion, and (d) smartphone usage. As a result, they found a representative received CSI waveform corresponding to smartphone usage (which involves several different movements: pick-up, move to front of face, look at phone, put back, hands back on the steering wheel). However, it turned out that the WiFi signal used was not from the smartphone. Consequently, both papers were excluded in Iteration 3.

**Table 4:** Statistics of the selection steps of the snowballing papers.

Researcher	Initial Analysis	Iteration 1	Iteration 2	Consolidation
1	Incl.: 232+57	Incl.: 19 + 2 Excl.: 213 + 55	Incl.: 16 + 2 Excl.: 216 + 55	
2	Incl.: 201 + 94	Incl.: 40 + 11 Excl.: 161 + 83	Incl.: 20 + 9 Excl.: 181 + 85	
3	Incl.: 0 + 397	Incl.: 0 + 135 Excl.: 0 + 262	Incl.: 0 + 11 Excl.: 0 + 386	
1, 2, and 3 (combined)	Incl.: 433 + 151	Incl.: 59 + 13 Excl.: 374 + 138	Incl.: 36 + 11 Excl.: 397 + 140	Incl.: 31 + 11 + 7 Excl.: 402 + 135

**Figure 2:**
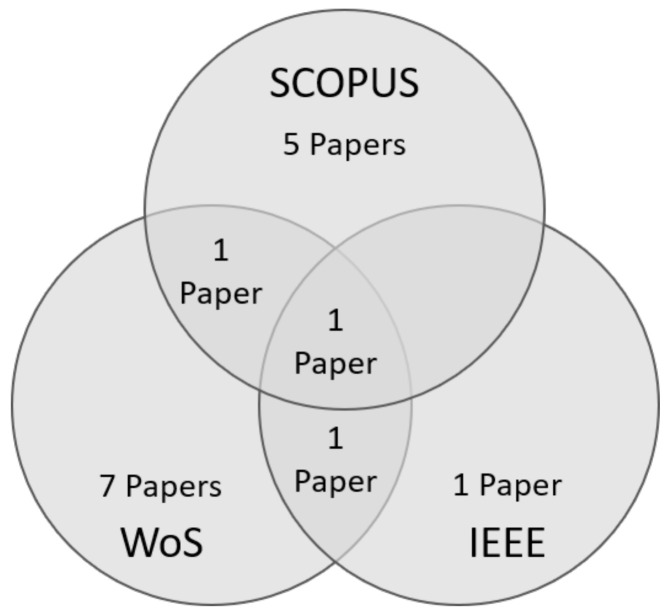
Selected papers and database where “Start set” papers were retrieved.

After the selecting step, 16 unique papers remained. These consisted of the so-called “Start set” papers (cf. Section 3.5). Figure 2 shows that amongst them are representatives of all three databases used. Two of the 16 unique papers can be found in two of the databases (Shabeer and Wahidabanu, 2012 [62], are in the result lists of Scopus and WoS, and Paruchuri and Kumar [63] are in the result lists of WoS and IEEE), while one (Song et al., 2016 [64]) is present in all three database result lists.

### 3.5. Snowballing

The method of snowballing was used to complement and enrich the research results, presented in the preliminary systematic literature review [23], with additional papers. These papers could not be found with the systematic literature review process executed since they did not come up in the search step. Snowballing serves as a valuable complement to the authors’ previous work [23] by effectively incorporating recent research findings that were challenging to identify through the systematic literature approach without the requirement of carrying out some steps in repetition. This method holds the potential to yield more pertinent results, with particular emphasis on the inclusion of updated or newer findings, especially with the forward snowballing search.

Snowballing refers to using the list of references from a set of papers, called the “Start set”, to identify additional papers. The start set we have used is considered a good set because it contains an adequate number of papers relevant to the topic originating from different communities (cf. [23], Table 3), as well as a good mix of journal and conference publications (44% and 56%, respectively). Furthermore, in the Start set, different authors are represented from a mix of countries (i.e., Canada, China, Egypt, Germany, Greece, India, Philippines, Singapore, and the USA). These add to the diversity of the Start set, which is important to have the necessary breadth of the research. The only aspect not adding to the diversity of the Start set is the institutions carrying out the research, which are mostly originating from academia (88%).

Two researchers executed the backward snowballing and then continued with forward snowballing. With backward snowballing, papers were collected from the list of references of the 16 selected papers that complied with the same set of inclusion/exclusion criteria mentioned earlier. In addition to them, the following criteria were used:

1. Exclude white papers, technical reports, and pre-prints;
2. Exclude press releases, annual reports, and factsheets;
3. Exclude links to products, software code, or datasets;
4. Exclude papers that were not about driver monitoring.

Then, with forward snowballing, the papers citing these papers were checked. Again, the same requirement was made, compliance with the inclusion/exclusion criteria.

In the initial analysis (cf. Table 4), two researchers performed backward and forward selections and initially looked at 433 backward-selected and 151 forward-selected papers. This process was carried out on 6 July 2021. Then, after two iterations where uncertain papers were discussed (see columns Iteration 1 and Iteration 2), the resulting papers were 36 and 11 (from the backward and forward process, respectively). A third researcher performed and updated forward selection on 24 May 2023 and added seven more papers. The factor that excluded most papers was the lack of smartphone usage as a device for driver behaviour detection. The papers were consolidated at the final consolidation step.

In the first iteration of the process, most of the papers were found clear to decide on whether to include or exclude, as they were irrelevant to the topic. In the cases where there was uncertainty, those papers were discussed. The titles and the abstracts of the papers were read and, in some cases, the whole paper. In the second iteration of the process, the researchers identified some papers that, at the first iteration, seemed relevant, for example, papers on risky driver behaviour, but which were either not using smartphone sensors or were on a broader topic, or even were not unique (in comparison to some other papers already included). These papers were spotted, discussed, and decided on one by one. That is, the researchers blindly inspected the papers and categorised them again as “include”, “exclude”, and “maybe”. The “maybe” or borderline papers were inspected by the researchers and then discussed in the consolidation step, and when necessary, the researchers resolved any conflicts they had, reaching a consensus. In many cases, the full papers needed to be read. In the consolidation step, all researchers discussed, in detail, all papers, after the selection, and also decided that very similar papers be removed at reporting (which had almost the same content but different publication venues; in such cases, the latest publication was kept) so that they were not over-represented in the analysis.

Then, three researchers went through all the papers together and added labels when a particular topic was investigated. The work was carried out during five workshops. The workshops had a two-hour duration.

## 4. Results

This section presents the reporting of the results based on an author-centric structure to answer RQs 1–3, given the new papers added from the authors’ previous work. The type of sensors used in each work is summarised in Table 5. The summaries of the studies are reported in a set of tables (cf. Table 6, Table 7, Table 8, Table 9 and Table 10).

Half (51%) of the papers are published in journals (appearing in Table 11) and the rest (49%) in conference venues (appearing in Table 12). The papers are scattered across multiple publication venues, and the combined number of papers in the top two venues only represents ca. 16% of the papers in total.

### 4.1. Author-Centric Analysis: Summary of Individual Results

This subsection presents the results of the author-centred analysis, including the research objectives of their contributions, the smartphone sensors used, the analysis method used, and the results obtained, in alphabetical order.

Ahn et al. [65] propose FuzzY inference (VERIFY), a system that recognises the vehicle-boarding directions solely using inertial measurement unit (IMU) sensors of smartphones. Using electromagnetic field (EMF) fluctuations, it detects when the smartphone is close to the vehicle, whether the person is entering from the left/right/rear/front entrance of the vehicle, and classifies the vehicle-boarding direction with a Fuzzy Inference System (FIS). The authors report that the proposed system achieves 91.1∼94.0% accuracy, outperforming the other methods compared by 26.9∼38.4% and maintains at least 87.8% accuracy, regardless of smartphone positions and vehicle types.

Ahn et al. [66] present a system capable of classifying the smartphone wearer into “driver” or “passenger” by classifying if they are sitting left or right (left–right classifier—LRC), front or rear (front–rear classifier—FRC), and if they have entered a vehicle (in-vehicle classifier—IVC). Thereby, it is “utilizing the inconsistency between gyroscope and magnetometer dynamics and the interplay between electromagnetic field emissions and engine startup vibrations”. In their method, they use the smartphones’ IMU data in a Bayesian classifier. They claim to identify the driver’s smartphone with 89.1% accuracy. However, the solution is limited, as the smartphone “should remain static while an engine is being turned on”.

Albert et al. [67] study the opinions of 37 experts through the Analytic Hierarchy Process (APH) on smartphone apps that have the greatest potential to reduce injury crashes. They refer to the following desirable types of smartphones: collision warning, texting prevention (both no-typing and no-reading), voice control (both text to speech and commands), and Green Box (in-vehicle data recorder—IVDR). Their results report which apps are less likely expected to be widely accepted and used, and which are to be expected to gain public support.

Alqudah et al. [68] classify different driving events using smartphone sensors (i.e., acceleration, gyro rotation, yaw, roll, pitch, rotation rate, quaternion, gravity, magnetic field, and orientation). They make use of different classification techniques, like Support Vector Machines (SVMs), decision trees, Discriminate Analysis, Naïve Bayes, k-nearest neighbour (KNN), and ensembles, and achieve an accuracy of 98% with decision trees.

Baheti et al. [69] use a dataset collected by Abouelnaga et al. (2018) [70] for distracted driver posture estimation and classified images to the following 10 classes: driving, texting on mobile phones using the right or left hand, talking on mobile phones using the right or left hand, adjusting the radio, eating or drinking, hair and makeup, reaching behind, and talking to a passenger. They use convolutional neural networks (CNNs) and report achieving 96.31% on the test set.

Bergasa et al. [54] present a system (DriveSafe) that uses computer vision and pattern recognition techniques on the smartphone to assess whether the driver is drowsy or distracted using the rear camera, the microphone, the inertial sensors, and the GPS. Distractions are evaluated with three different indicators (acceleration, braking, and turning), and drowsiness as well as a distraction scores are calculated. Lane weaving and drifting behaviours are measured to infer drowsiness, whereas distractions are based on sudden longitudinal and transversal movements. Data from 12 drivers in two different studies are used to detect inattentive driving behaviours, obtaining an overall precision of 82% at 92% of recall.

Berri et al. [71] present an algorithm that allows the extraction of features from images to detect the use of mobile phones by drivers in a car. The experiments are performed on a set of images containing 100 positive images (people using phones) and 100 negative images (people not using phones). SVM and its kernels are tested as candidates to solve the problem. Tests on videos show that it is possible to use image datasets for training classifiers in real situations. A polynomial kernel (SVM) is the most advantageous classification system with an average accuracy of 91.57% for the set of images analysed.

Bo et al. [72] distinguish between drivers and passengers, and detect texting using smartphones, based on irregularities and rich microphone movements of users. The approach is based on the observation that the majority of drivers carry their phones in their trousers’ pockets, and they extrapolate that they enter a vehicle by a leg-lifting movement (the direction of turning and sequence), thus distinguishing entering a vehicle from the left or right. Then, they distinguish front or back seat positioning based on the vibrations felt when wheels hit bumps or potholes. The authors consider both the time interval between typing multiple sentences on smartphones and the frequency of the typing to discern between no driving and driving (aka distraction) scenarios. Using the Hidden Markov Model (HMM), they report a classification accuracy of 87% and a precision of 96.67% with 20 different typing cases of non-driving and driving in the parking lot.

Bortnik et al. [73] present an approach to detect if the smartphone has been used while driving without accessing personal data. This driver distraction approach makes use of Android dumpsys diagnostic data and detects online activities like social media, calling, and texting, as well as offline activities like taking pictures and browsing media. A synthetic case study is conducted and shows the ability of this approach to help, e.g., police officers to examine driver distraction in a car accident investigation.

Caird et al. [74] provide a meta-study on the topic of driving and texting (reading and typing), where the results from 28 studies are quantitatively presented and compared.

Castignani et al. [75] develop a smartphone-based event detection approach to identify driving manoeuvres related to the driving style (calm or risky). They use a fuzzy system that calculates a score for different drivers based on real-time contextual information, such as route topology or weather conditions. The driver starts a trip with 100 points, but when a driving-related event, such as hard braking, hard acceleration, over-speeding, or aggressive steering, occurs, the driver loses points depending on the type and severity of the event and its context (i.e., weather conditions and time of day). Similarly, the score is increased again if no event occurs during 0.5 km of driving. In an evaluation study with ten different drivers along a predefined route, an accuracy of more than 90% in event detection is achieved when the calibration time is at least 17 min and the distance travelled is 9.21 km.

Chen et al. [76] develop algorithms for detecting and differentiating various vehicle steering patterns, such as lane change, turn, and driving on curvy roads using non-vision sensors on smartphones. The performance of the smartphone-based prototype system is evaluated with a longer road test containing various road features, achieving 100% accuracy in detecting both right and left turns, regardless of the phone’s placement and road condition, 93% accuracy for lane changes when the phone is mounted on the windshield, 85% accuracy for lance changes when the phone is in the driver’s pocket, nearly 97% accuracy in detecting curvy roads with the phone mounted on the windshield, and nearly 92% accuracy with the phone kept in the driver’s pocket.

Chu et al. [77] develop a set of ML-based algorithms to detect whether a smartphone user is a driver or passenger in a vehicle. The basic idea is to detect micro-activities (entry swing, seatbelt use, and pedal press) and event triggers (walking and pause, vehicle motion, and timeouts) using smartphone sensors, which, in turn, allows for the distinction between driver and passenger. Their first prototypes, running on Android and iOS operating systems and tested with six users in two different vehicles, achieved overall recognition accuracies of over 85%.

Chuang et al. [78] intended to estimate driver gaze direction to detect driver distraction. In their online classifier approach, they record videos with the front camera of a smartphone placed in front of the driver. In the first step, the head pose is detected and delivers features for the eight-class gaze classifier. Classification is conducted with a multi-class linear support vector machine (SVM) classifier. Due to the poor generalisation performance of the online classifier training, offline classifier training with an in situ approach is performed. Therefore, videos are collected only once for a given setup of driver, vehicle, and camera position. Four experimental scenarios with four different training techniques are tested, leading to the conclusion that the proposed training technique comes close to the chosen standard. Splitting the eight classes from the gaze classifier in safe and unsafe driver behaviour, the classifier tested on the four experiments shows classification accuracies between 86.4% and 97.4%.

Dai et al. [79] identify the driver’s talking direction, namely, front, right (when the driver is sitting on the left side), and back, from running vehicles. They use two microphones on a smartphone and a K-means clustering algorithm to first identify whether the driver is talking or not and then classify the sound into one of the three driver talking directions. The algorithm performs 95% accuracy on average for four different smartphone placements, at least 92.2% accuracy on three specific scenarios (i.e., garage campus, downtown, and windows opened or closed) and 90.3% accuracy when the window is opened, i.e., when there is the presence of noise from outside the vehicle. The results are based on 23 collected hours of voice data from 20 participants, using two brands of phones and two vehicles.

Dua et al. [49] develop an ML-based system that uses the front camera of a windshield-mounted smartphone to monitor and rate driver attention by combining multiple features based on the driver state and behaviour, such as head pose, eye gaze, eye closure, yawns, and the use of cell phones. Ratings include inattentive driving, highly distracted driving, moderately distracted driving, slightly distracted driving, and attentive driving. The evaluation with a real-world dataset of 30 different drivers showed that the automatically generated driver inattention rating has an overall agreement of 87% with the ratings of five human annotators for the static dataset.

Dua et al. [49] aim to identify driver distractions using facial features (head pose, eye gaze, eye closure, yawns, the use of smartphones, etc.). The smartphone’s front camera is used as well as three approaches: in the first, convolutional neural networks (CNNs) are used to extract the generic features and then a gated recurrent unit (GRU) is applied to obtain a final representation of an entire video. In the second approach, besides having the features from a CNN, they also have other specific features, which are then combined using a GRU to obtain an overall feature vector for the video. In the third approach, they use an attention layer after applying long short-term memory (LSTM) to both specific and facial features. Their automatically generated rating has an overall agreement of 88% with the ratings provided by five human annotators on a static dataset, whereas their attention-based model (third approach) outperforms the other models by 10% accuracy on the extended dataset.

Eraqi et al. [50] aim to detect ten types of driver distractions from images showing the driver. They use (in one phase) the rear camera of a fixed smartphone to collect RGB images, in order to extract the following classes with convolutional neural networks (CNNs): safe driving, phone right, phone left, text right, text left, adjusting radio, drinking, hair or makeup, reaching behind, and talking to passenger. Thereby, they run a face detector, a hand detector, and a skin segmenter against each frame. For the results, first, they present a new public dataset, and second, their driver distraction detection solution performs with an accuracy of 90%.

Gelmini et al. [46] make use of the smartphone for four-dimensional driving-style risk assessment, based on inertial sensors included on the smartphone. The approach to assessing risk is based on the following four cost functions: speed (relating the measured speed to the ideal speed for a specific road), longitudinal acceleration (analysing the driving behaviour during any speed variation), lateral dynamics (considering the yaw rate or speed when the driver is changing direction), and smartphone hand usage. They apply the use of thresholds to detect the use of the smartphone, based only on acceleration and angular velocity measurements, whereas the authors support that other methods such as deep learning or ensembles could be alternatively explored. Their results indicate simply “safer” as opposed to “less safe” driver profiles, whereas data are collected from over 5000 kilometres of varied car trips.

He et al. [80] develop a system to determine the location of smartphones in a vehicle on a seat level (front/back and left/right) only using embedded sensors. To detect if a smartphone is left or right from another phone in a vehicle, He et al. [80] use the centripetal acceleration in their algorithm, combined with techniques for synchronisation and amplitude calibration. The main idea is that in the event of a turn, the acceleration differs according to the position of the car (left/right). To identify whether a phone is in front of the car or in the back is performed with the help of the vertical acceleration signals. In the event of an uneven surface, the vertical acceleration differs in time. He et al. [80] combined this basic idea with calibration, re-sampling, and sliding-window techniques to create an algorithm to detect the front or back position of a smartphone. Comparing different experiments (four different smartphones, two different cars, and ten different positions), positioning accuracy reached between 70% and 90% in real city environments.

Hong et al. [81] build an In-Vehicle Smartphone-based Sensing Platform (IV-SP2) to assess if a person has an aggressive driving style. They choose two methods for finding the ground truth: one is based on self-reports of accidents and speeding tickets and the other is based on the Manchester Driving Behaviour Questionnaire (DBQ) and some additional questions concerning aggressive driving. Data are collected with 22 drivers over 3 weeks, leading to 1017 trips and 542 hours of data in total. Hong et al. [81] create three naïve Bayes classifiers for each ground truth using different combinations of sensor data. Model 1 is a smartphone-only model, Model 2 adds a Bluetooth-based on-board diagnostic (OBD2) reader, and Model 3 also includes an inertial measurement unit (IMU). Model 3 performs best with 90.5% accuracy for the self-report method and 81% accuracy for the questionnaire method. Model 1, using only smartphone sensors and no data about the car, has an accuracy of 66.7% for both methods.

Janveja et al. [52] present a smartphone-based system to detect driver fatigue (based on eye blinks and yawn frequency) and driver distraction (based on mirror scanning behaviour) under low-light conditions. In detail, two approaches are presented—in the first, a thermal image from the smartphone RGB camera is synthesised with a generative adversarial network, and in the second, a low-cost near-IR (NIR) LED is attached to the smartphone—to improve driver monitoring under low-light conditions. For distraction detection, statistics are calculated if the driver is scanning their mirrors at least once every 10 s continuously during the drive. A comparison of the two approaches reveals that the “results from NIR imagery outperforms synthesised thermal images across all detectors (face detection, facial landmarks, fatigue, and distraction).” As a result, they mention 93.8% accuracy in detecting driver distraction using the second approach, the NIR LED setup.

Jiao et al. [82] introduce a hybrid deep learning model to detect different distracted driver actions as well as a new dataset. The dataset was created with a smartphone placed in the right upper corner of the vehicle and covered eight different actions (e.g., safe driving, eating while driving, drinking while driving, or talking with passengers). The model consists of four different modules. The first module estimates the human body pose (OpenPose), the second module processes the data and constructs features, the third module extracts keyframes (K-means), and the last module recognises actions (LSTM). After experimenting with different combinations of modules and features as well as tuning hyperparameters, the best model shows an accuracy of 92.13%.

Johnson et al. [83] present the MIROAD system that uses the dynamic time warping (DTW) algorithm for classifying driver behaviour based on a set of events, like left and right manoeuvres, turns, lane changes, device removal, and excessive speed and braking. The data collection and processing is carried out on a smartphone. The method also uses the Euler representation of device attitude. The system also produces audible feedback if a driver’s style becomes aggressive, as well as the information leading up to an aggressive event, including video, location, speed, and recent driving manoeuvres. The results presented refer to driving events mostly, i.e., the U-turn was correctly identified 23% of the time (using the accelerometer) and 46% of the time (using the gyroscope); 77% of the cases, the U-turn was correctly classified, whereas 97% of the aggressive events were correctly identified.

Kapoor et al. [48] design a system capable of detecting distracting tasks by the classification of driver images through a pre-trained convolutional neural network (CNN) model(s). Driver images of the “custom dataset” are taken from the smartphone camera, and the CNN models can even run within the constraints of an Android smartphone. Thus, “the system is designed to distinguish the state of the driver in real-time using only an Android phone (mounted on vehicle dashboard) without any need of additional hardware or instruments in the vehicle.” In the case of a detected distraction, an alert is generated with a beep sound. The ten classes of distraction are taken from the State Farm Distracted Driver dataset, which is used for fine-tuning the CNN models. Finally, they state an accuracy between 98 and 100% for four classes (e.g., calling or texting on mobile), if they fine-tune with public datasets.

Kashevnik et al. [5] present a methodology for the creation of a multimodal corpus for audio-visual speech recognition using smartphones. They consider use cases that require speech recognition in a vehicle cabin for interaction with a driver monitoring system and use cases where the system detects dangerous states of driver drowsiness and starts a question–answer game to prevent dangerous situations. Finally, based on the proposed methodology, they develop a mobile application that allows them to record a corpus for the Russian language.

Khurana and Goel [84] detect phone usage of drivers using on-device cameras. Thereby, they present a software-based solution that uses smartphone camera images to observe the vehicle’s interior geometry and detect the phone’s position and orientation. For model training, they use continuous video recording to obtain a large dataset of images. In addition, they use IMU sensors (accelerometer and gyroscope) to detect if the phone is docked; however, this is not described in detail. The authors’ system is able to distinguish between driver and passenger use of the phone. The authors train random forest classifiers on data collected in 16 different cars from 33 different drivers and claim to have achieved an overall detection accuracy of about 90 % to distinguish between the driver and passenger. Thereby, the phones can be held by the persons or mounted on a docking station. However, it is not possible to collect data for the phone in the in-hand position in real-time.

Koukoumidis et al. [85] use a windshield-mounted phone to detect current traffic signals with its camera, collaboratively communicate and learn traffic signal schedule patterns, and predict their future schedule. They apply image processing techniques such as colour filtering, edge detection, and Hough transformation, and use colour confidence intervals of candidate areas in video images to detect traffic signals. In addition, machine learning (Support Vector Regression) is applied to predict the traffic light schedule, IMU sensors are used to infer the orientation of the traffic light and improve the detection quality, and GPS is used to calculate the distance to the signal. The evaluation for two different deployment scenarios shows that the system correctly detects the presence (or absence) of a traffic signal in 92.2% and 87.6% of cases, respectively. The timings for the preset traffic signals are predicted with an average prediction error of only 0.66 s and those for the traffic-adapted traffic signals with an error of 2.45 s.

Li et al. [86] combine different data from smartphones and WiFi signals to identify 15 risky driving actions (e.g., snoring, head turned, the use of phones, hands on the steering wheel). The system called WisDriver classifies risky driving actions into three types: head movement, arm movement, and body movement. It uses the wireless signal information (Channel State Information—CSI) to identify the driver’s posture, and together with the built-in smartphone sensors (such as accelerometers, gyroscopes, and magnetometer), it is used to detect the vehicle’s status (speed and direction). The approach is field-tested on 20 drivers and indicates an accuracy of 92% in identifying dangerous driving behaviours.

Lindqvist and Hong [87] conduct user-interaction-related research for the design of driver-friendly mobile phone systems that do not distract drivers. They present initial interaction designs for a mobile phone system that has the potential to encourage people not to use their mobile phones while driving. They use different concepts such as context awareness for burden shifting from caller to call recipient, time shifting and activity-based sharing to address the mobile information needs of drivers and the people who might call them. Their core idea is that drivers will not be distracted by their mobile phones unless someone they know and trust is calling in an emergency.

Liu et al. [88] present a system that can recognise internal driver inputs such as steering wheel angle, vehicle speed and acceleration, and external perceptions of the road environment (e.g., road conditions and front view video) using a smartphone and an IMU mounted in a vehicle. The accuracy is assessed using more than 140 trips collected over a three-month period. The steering wheel angle is estimated with a mean error of 0.69, the vehicle speed is derived with a deviation of 0.65 km/h, and binary road conditions are estimated with 95% accuracy.

Ma et al. [89] propose a scheme for identifying dangerous driving behaviour and have developed an algorithm for the automatic calibration of smartphones based on the determination of the sensor noise distribution when a vehicle is being driven. Their system uses the corrected sensor parameters to identify three types of dangerous behaviours: speeding, irregular direction change, and abnormal speed control. They evaluate the effectiveness of their system in realistic environments and find that, on average, it is able to detect the events of driving direction change and abnormal speed control with 93.95% accuracy and 90.54% recall, respectively. In addition, the speed estimation error of their system is less than 2.1 m/s.

Mantouka et al. [90] use data collected from smartphone sensors to identify unsafe driving styles based on a two-stage K-means clustering approach and use information on the occurrence of harsh events, acceleration profiles, mobile phone use, and speeding. Trips where the driver uses the smartphone are classified as distracted trips. Variables used are harsh acceleration and hard brakes per km, a smoothness indicator, the standard deviation of acceleration, the percentage of mobile phone use, and the percentage of speeding. In the first clustering, the authors separate aggressive from non-aggressive trips, while in the second clustering, they distinguish normal trips from unsafe trips. Finally, the trips are categorised into six groups: aggressive trips (aggressive trips, distracted trips, and risky trips) and non-aggressive trips (similar: safe trips, distracted trips, and risky trips). The authors claim that 75% of the 10,000 recorded trips (from 129 drivers) did not have aggressive features, and in just 8% of the trips, the driver was actually distracted.

Mantouka et al., in a follow-up publication [91], present a driving recommendation framework for improving the driving behaviour of individuals regarding driving aggressiveness and riskiness. The data used in the development are recorded with a smartphone app during 153,953 trips from 696 distinct drivers. Two different levels are considered within the approach: trip (specific trip) and user level (overall driving behaviour). For each level, a reinforcement learning (RL) controller based on the deep deterministic policy gradient algorithm (DDPG) is created. The results in a microscopic simulation using Athens’s road network show that it would lead to safer and less aggressive driving, but the traffic conditions, in general, do not improve.

Meiring et al. [92] investigate driving style analysis solutions and the machine learning and artificial intelligence algorithms used. The following driving styles are described: normal/safe, aggressive, inattentive, and drunk driving, as well as driver fatigue and driver distraction. They identify and describe several fields of applications for assessing driver styles, for example, driver assistance, drowsiness detection, distraction detection, early warning applications, accident detection, and insurance applications. Meiring et al. [92] elaborate on the most popular algorithms and identify fuzzy logic inference systems, hidden Markov models, and Support Vector Machines to be of special interest in the future.

Meng et al. [93] introduce the system OmniView, which helps the driver to be aware of all surrounding vehicles. OmniView uses smartphones and their cameras to compute a map with the relative positions of all the vehicles next to a car. Communication is conducted via Dedicated Short-Range Communication (DSRC) and vehicles identify each other with an image of themselves. The system includes five functional parts: vehicle detection, vehicular communication, image matching, position calculation, and map computation. Meng et al. [93] evaluate all these parts separately either by actual testing/calculation or simulation. They conclude that OmniView might be able to create a map of the surrounding vehicles in real-time and assist drivers.

Mihai et al. [53] describe an approach to create an estimator for head orientation in the automotive setting using the front camera of a smartphone. They test several smartphones, implement face detection and head orientation detection on an iPhone 6, and carry out a case study with two scenarios. The first scenario includes a real vehicle and tests subjects performing specific tasks to obtain the head orientation detection running (calibration procedure). The second scenario is conducted in an ECA (https://www.ecagroup.com/ accessed on 24 July 2023) Faros Simulator. Test subjects drive in an urban environment, and sound alarms go off when no face or no head orientation is detected. The collected data indicate that some more work must be conducted, i.e., the head orientation has to be aligned with the coordinate system of the smartphone.

Nambi et al. [94] develop Harnessing Auto-Mobiles for Safety (HAMS), a smartphone-based system to monitor drivers and driving. The driver monitoring system uses the front camera of a smartphone and detects driver drowsiness, driver distraction, and the driver’s gaze. The basis for these detection tasks is the localisation of facial landmarks. These landmarks are combined with different metrics to detect, i.e., eye closure. For driving distraction, a pre-trained model is fine-tuned to detect if a driver is talking on the phone. Gaze detection is conducted with OpenCV-implemented algorithms like Perspective-n-Point and Random Sample Consensus together with the LeNet-5 network. The back camera of a smartphone is used to monitor the actual driving. Two tasks are implemented: vehicle ranging and lane detection. Vehicle ranging uses a deep neural network (DNN) to estimate the distance to the vehicle in front. A three-way lane classifier with the help of a pre-trained AlexNet and a support vector machine (SVM) is built for the actual classification. The HAMS system is implemented as an Android app and is tested on two smartphones.

Omerustaoglu et al. [51] integrate sensor data into vision-based distraction detection models to improve the performance of distraction detection systems. They construct a two-stage distracted driving detection system to detect nine distracting behaviours using vision-based convolutional neural network (CNN) models and long short-term memory–recurrent neural network (LSTM-RNN) models using sensor and image data together. Specifically, both hybrid and predictive level fusion increased overall accuracy by 9%, from 76% to 85%, compared to using image data alone. They also found that using sensor data increased the accuracy of detecting normal driving from 74% to 85%.

Othman et al. [95] collect data from 633 different drivers to create an extensive dataset for driver monitoring and behaviour analysis. Smartphones are used to gather data with their embedded accelerometer, gyroscope, and magnetometer. In addition, the mobile devices recorded videos which are then fed into deep neural network models to obtain features, like driver’s head pose, safety belt state, and mouth-openness ratio, to detect dangerous states. A smartwatch is also used to obtain the heart rate of the driver. Data evaluation is conducted with an unsupervised learning approach (K-means) to detect clusters in certain features regarding critical events. The results show that drivers tend to not use seat belts in cities, and drowsiness is more common on highways.

Pargal et al. [96] introduce an approach to let a smartphone detect if it is used while driving and who uses it (driver, passenger). The idea behind this is that drivers want to use the phone while driving and use methods to fool systems that recognise driving with the help of, e.g., cameras or Bluetooth. The authors, therefore, propose a blind approach, only using the ambient mechanical noises within the car for detection. The final single-step algorithm shows F1 scores from 0.75 to 0.875 for different smartphone placement scenarios, although the authors state that there are still some issues to make the system robust.

Park et al. [97] present an Automatic Identification of Driver’s Smartphone (AIDS) system, which uses smartphone sensor information to identify the position and direction of the smartphone, as well as vehicle-riding activities, such as walking towards the vehicle, standing near the vehicle while opening a vehicle door, entering the vehicle, closing the door, and starting the engine. Entering a vehicle is detected by analysing electromagnetic field (EMF) fluctuations, significant vertical accelerations caused by sitting-down motions, and vehicle door closing sounds (VDCSs). Vehicle entering directions (left or right) are differentiated by analysing the body rotations using EMF. Seated (front or rear) rows are differentiated by analysing subtle EMF changes monitored when starting the engine. The results using seven different vehicles show that entering a vehicle is detected with a 90–93% true positive rate (TPR) and a 91–93% true negative rate (TNR), while entering directions are identified with an 87–95% TPR and an 84–90% TNR. Moreover, TPR and TNR of seated row classification results are found to be 82–99% and 79–95%, respectively. Finally, AIDS identifies the driver’s phone with an 83–93% TPR, while the TNR is 90–91%.

Paruchuri and Kumar [63] present how a smartphone camera can be used to provide context and/or the position of the smartphone. The paper focuses on distinguishing the driver from the passengers by comparing images from the smartphone camera to reference images. In particular, they compare the angle difference of reference objects (e.g., ventilation grille) and calculate the distance between images to locate the phone position. As a result, unfortunately, 15 out of 38 images are registered incorrectly.

Punay et al. [98] present a summary of the “unDivided” mobile application, which utilises GPS data to calculate vehicle speed and warns when certain speed limits are exceeded. The application auto-starts when driving is detected (the speed of human running/walking is exceeded). When driving is detected, the application automatically turns down and answers back with messages when the driver receives calls or messages, while it allows emergency calls to go through, to keep distraction to the necessary minimum. In addition, it tracks the users’ location, provides navigation features, and implements e-call functionalities. However, no evaluation is available, as the system seems to be in a prototypical state.

Qi et al. [99] present a human activity detection system for two areas inside the vehicle based on audio information (chatting, silence, etc.) and context information (clear or crowded traffic) for areas outside the vehicle, which is derived from IMU-based vehicle dynamics detection (brakes, lane changes, turns, and stops). Inside the vehicle, a microphone is used to record audio and infer human activities. For context information, they used their (IMU- and GPS-based) activity detection methods with a convolutional-neural-network-based model to derive a data fusion model (including OBD-II data) for activity detection. They report 90% detection accuracy for seven different activities by combining data from multiple sensors.

Qi et al. [100] present a system called DrivAid, which collects and analyses visual and audio signals in real-time, as well as data from IMU and GPS sensors to detect driving events. The following events are of interest: (i) detecting vehicles, people, traffic signs and speed limits from the front view camera; (ii) detecting vehicles and people from left and right blind spot cameras; (iii) estimating head poses from the face camera; and (iv) monitoring turn signal usage from audio streams. The system uses computer vision techniques on a vehicle-based edge computing platform to complement the signals from traditional motion sensors. Using deep learning inference, an average of 90% event detection accuracy is achieved.

Rachmadi et al. [101] propose a driver abnormal behaviour detection system that uses only two sensors from a smartphone (accelerometer and gyroscope). With this data, four normal and five dangerous driving behaviours of motorcycle drivers can be detected with the help of a multi-layer perceptron (MLP). Normal behaviours are, e.g., turning left/right and going straight; dangerous actions include, e.g., sudden acceleration or braking. The dataset is created with the help of five different motorcycle riders; the sampling rate is 5 s with a sampling window of 100 ms. The architecture is divided into three common parts: (i) data reading, (ii) data processing and training phase, and (iii) model evaluation. The best model shows an accuracy of 97.5% and takes 45 ms for calculation.

Shabeer and Wahidabanu [62] detect incoming or outgoing phone calls while driving using an antenna located on the top of the driver’s seat for detecting when the driver uses their mobile phone. Thereby, a GSM signal connection between the smartphone and other entities of the GSM Network Architecture (e.g., mobile switching centres and base stations with associated base transceivers) is detected. If a call is detected, a low-range mobile jammer is used to prevent drivers from receiving base station signals, with its range covering only the driver seat. However, no evaluation is available, as the system seems to be in a prototypical state.

Singh et al. [102] do not detect driver distraction itself but develop a system that alerts the driver in case of detected vehicles in a blind spot, thus assisting the driver if they are distracted. In particular, the developed smartphone-based system monitors the blind spot on the driver side in real-time and alerts the driver about the presence of a vehicle. Using images from the smartphone’s front camera, two approaches are explored based on intensity variation and contour matching to detect a vehicle in the blind spot. They state that their system is able to detect vehicles in the blind spot with an accuracy of 87% in real-time and warn the driver accordingly.

Song et al. [64] detect driver phone calls by using audio and voice recognition, combined with the smartphone’s call state. Thereby, they use a client–server-based system with smartphones being the clients, which is extended with a unidirectional microphone and placed in front of the driver seat, together with an on-board unit being the server. They state that the system is able to use the driver’s voice features to differentiate a driver from other passengers, thus also determining whether the driver is participating in a current phone call or not. In particular, first, they collect the driver’s audio signals for training; second, transform them into feature vectors by feature extraction; and third, train a speaker model using the driver’s feature vectors. The detection system will cut off the phone call if the similarity score is higher than a certain threshold. An evaluation shows that the system’s true positive rate (TPR) is above 98% for three different evaluated passenger positions, over 90% with the impact of noise, 80% if three people are talking, and 67% if four people are talking.

Torres et al. [103] propose a non-intrusive technique that uses only data from smartphone sensors and machine learning to automatically distinguish between drivers and passengers when reading a message in a vehicle. They evaluate seven machine learning techniques in different scenarios and find that convolutional neural networks (CNNs) and gradient boosting are the models with the best results in our experiments. Their results show accuracy, precision, recall, F1 score, and kappa metrics above 0.95.

Tortora et al. [104] present an Android application to detect driver inattention using embedded sensors in the smartphone. The following distractions can be noticed: drowsiness, turned head, smartphone usage, and smartphone falls, as well as excessive noise. Combining these distractions with the speed of the car and the tortuosity of the road, an index is calculated that represents the level of inattention. Including both speed and tortuosity is based on the idea that these factors make inattention more dangerous. This “distraction score” is visible to the user via a coloured bar in the Android application. The calculation of the score is in real-time, no sensitive data are stored, and only a summary of each trip is stored on the smartphone.

Tselentis et al. [105] provide a structured approach to studying the evolution of driving efficiency over time, with the aim of drawing conclusions about different existing driving patterns. They base their work on a dataset that uses smartphone device sensors during a naturalistic driving experiment in which the driving behaviour of a sample of two hundred drivers is continuously recorded in real-time over 7 months. Their main driving behaviour analytics considered for the driving assessment include distance travelled, acceleration, braking, speed, and smartphone usage. Their analysis is performed using statistical, optimisation and machine learning techniques. They use K-means for clustering, resulting in three main driver groups: moderate drivers, unstable drivers, and cautious drivers.

Wang et al. [106] present an approach based on smartphone sensing of vehicle dynamics to determine driver phone use. Thereby, they use smartphone sensors, i.e., accelerometers and gyroscopes, to detect differences in centripetal acceleration due to vehicle dynamics using a simple plug-in module for the cigarette lighter or OBD-II port. These differences combined with angular speed can determine whether the phone is on the left or right side of the vehicle. The experiments conducted with two vehicles in two different cities demonstrate that the system is robust in real driving environments. Their system can achieve a classification accuracy of over 90%, with a false positive rate of a few per cent. They also find that by combining sensing results in a few turns, they can achieve better accuracy with a lower false positive rate.

Vasey et al. [107] aim to address the impact of driver emotions, such as anger and happiness, on driving behaviour and driver distraction. Thus, they describe a system to identify a driver’s emotional arousal. Thereby, they make use of an Android application as a hub to collect data from the driver’s physiological and the vehicle’s kinematic data. The smartphone’s accelerometer, jerk, and GPS data; a wearable chest band sensor to collect the driver’s heart rate; and a vehicle’s OBD-II connector to read CAN bus data, i.e., accelerator pedal position, steering wheel angle, and engine RPM, are used. They mention a machine learning classifier, such as a decision tree, support vector machine (SVM), and neural network will be used to train. Questionnaires are planned to be used to rate the driver’s emotional state and workload. However, the paper presents a concept, and there are no results in it.

Vlahogianni and Barmpounakis [108] propose a device reorientation algorithm, which leverages gyroscope, accelerometer, and GPS information, to correct the raw accelerometer data, and use a machine learning framework based on rough set theory to identify rules and detect critical patterns solely based on the corrected accelerometer data. They use their approach to detect driving events (such as braking, acceleration, and left and right cornering), so they do not report any results related to distraction. Their results, based on data collected using a fixed-position device, compare the use of OBD-II and smartphone devices. The total accuracies for the smartphone and OBD-II devices are 99.4% and 99.3% respectively. TPRs (sensitivity) are 88.1% and 86.6%, while the FPRs are 0.3% and 0.4% for the smartphone and the OBD-II devices, respectively.

Woo and Kulic [109] propose a classifier-based approach for driving manoeuvre recognition from mobile phone data using SVMs. They investigate the performance of a sliding window of velocity and angular velocity signals obtained from a smartphone as features, using principal component analysis (PCA) for dimensionality reduction. The classifiers use simulated vehicle data as training data and experimental data for validation. Classifier performance was achieved with an average precision of 81.58% and an average recall of 82.79%, resulting in an average F1 score of 81.94%. The balanced accuracy was calculated to be 88.74%.

Xie and Zhu [110] compare three window-based feature extraction methods for driving manoeuvre classification, statistical values, and automatically extracted features using principal component analysis and stacked sparse auto-encoders. First, after pre-processing, they segment all sensor information from each dataset into windowed signals. Then, they apply three feature extraction methods to these windowed signals and, finally, feed the extracted features into a random forest classifier. The performance of their manoeuvre classification is evaluated on three different datasets and shows weighted classification F1 values of 68.56%, 80.87%, and 87.26%. Statistical features perform best on all three datasets.

Xiao and Feng [111] describe a driver attention detection system based on smartphones with dual cameras. It consists of three modules: the first module is an estimator of gaze direction (pupil location, yaw and pitch angles of eyes, and the position and size of face detected with the smartphone front camera), the second is a detector of road motion objects, and the third module is an inference engine that integrates the input from the above-mentioned two modules and outputs a voice alert to the driver when needed. An SVM classifier is used to estimate the gaze area, and Lucas–Kanade optical flow is used to detect road motion objects combined with dynamic background compensation. As a result, they state 93% accuracy for gaze estimation and 92% overall accuracy.

Xie et al. [112] compare the utility of different features for driving manoeuvre classification. The data are collected from the readings of accelerometers, gyroscopes, and GPS sensors built into smartphones. They first extract features using PCA and stacked sparse auto-encoder (SSAE) using windowed data. Then, statistical features are compared to the PCA- and SSAE-based features before driving manoeuvre classification using a random forest classifier is performed. They compare the three feature extraction methods using three datasets provided by Intelligent Mechatronics Systems Inc (IMS), University of Alcalá (UAH), and University of Waterloo (UW) and consist of different drivers, road conditions, phone locations, and sample rates. They report weighted classification performance of F1 scores of 68%, 80%, and 87% on three different datasets.

Xie et al. [113] develop a smartphone-sensor-based driver distraction system using GPS and IMU data and an ensemble learning method to detect vehicle shifting and erratic braking for instance. Ensemble learning of four standard classifiers is used, namely, k-nearest neighbour (KNN), Logistic Regression, Gaussian Naive Bayes, and random forest. They state that their “best-performing model can achieve a weighted F1 score of 87% using all signals”.

Yang et al. [114] detect smartphone usage in cars to distinguish between a driver and a passenger using a smartphone by estimating the range between the phone and the car’s speakers. The method classifies the location of the smartphone using the car’s stereo system, the Bluetooth connection, and the smartphone’s speaker and microphone. The method relies on threshold-based classification. Experimentation is carried out with two types of smartphones and two types of cars, and the classification accuracy is obtained with calibrated thresholds, the detection rate is above 90%, and the accuracy is around 95%.

Yaswanth et al. [115] consider a sequence of actions that trigger the identification of smartphone detectors as follows: walking–standing–entering–seated–engine starts. The following detectors are used: entering direction classifier (EDC), walking and standing detector (WSD), entrance detector (ETD), seated row classifier (SRC), and smartphone position classifier (SPC). SRC checks whether the driver is in the seat or not. SPC distinguishes between three frequent positions used by the user to hold the smartphone: pockets, bags, and hands. The system uses electromagnetic spikes triggered by the actions above and the engine starts. In order to save energy, accelerometer and magnetometer readings are used to detect if the driver has finished entering the vehicle; thus, other sensors are woken up. However, the paper presents a concept, and there is no evaluation available.

You et al. [116] present a system (CarSafe) based on computer vision and machine learning algorithms operating on the phone to monitor and detect whether the driver is tired or distracted using a front-facing camera, while, at the same time, tracking road conditions using a rear-facing camera. With respect to distraction, two-phase decision trees are used to classify face direction and SVMs are used to classify eye states. The precision obtained for face direction to the right is 68% and recall is 68%, and for face direction to the left, it is 79% and 88%, respectively. The accuracy, precision, and false positive rates of eye state classification (open–closed) are 92%, 93%, and 18%, respectively.

Ziakopoulos et al. [117] investigate the reasons that driver distraction through smartphone usage happens while driving. They conducted an experiment with 230 drivers having a non-intrusive driving recording application installed. Drivers go through six different phases, starting with no feedback on the app, leading to showing a scorecard for safe driving on the phone, including maps, displaying comparisons between the driver and other drivers, and back to no feedback. These data were completed with additional self-reported questionnaire data and resulted in data from 50,728 trips and 87 drivers, which were then analysed with XGBoost algorithms. The results showed that the reasons for drivers using smartphones involved a number of complex relationships, such as an increase in total trip distance, the number of tickets in recent years, etc., leading to less phone usage. On the other hand, older drivers or driving more kilometres annually leads to more phone usage.

### 4.2. Aggregated Results: Smartphone-Based Approaches for Driver Distraction Detection

**Figure 3:**
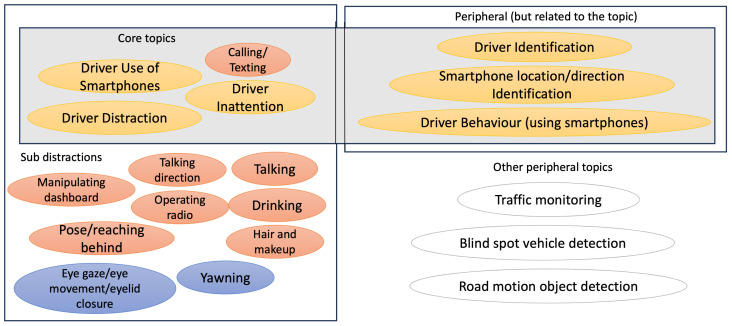
Core topics related to driver distraction, driver inattention, driver use of smartphones, and their subtopics.

The papers identified on monitoring distracted driving behaviour using smartphones can be categorised into several core topics: Firstly, there are papers that focus on driver distraction, driver inattention, and the driver’s use of smartphones. Related to this category, some papers specifically address the subtopic of calling or texting while driving. A visualisation of the core topics and the subtopics related to driver distraction were found in the papers and are shown in Figure 3.

Several papers tackle problems that are still relevant to the topic under analysis, for example, identifying whether the driver or a passenger is using the smartphone and determining the location or direction of the smartphone during the driving activity. These are peripheral yet significant problems to the core topic. Moreover, a lot of papers that investigate driver behaviour patterns associated with risky driving behaviours, such as speeding or changing lanes, were discovered. These studies provide valuable insights into the relationship between driver behaviour and distracted driving.

Several other subtopics related to driver distraction were investigated in the reviewed papers, too. These included examining various actions while driving, i.e., examining the direction of talking, talking/drinking, examining eye gaze patterns, identifying eye movements, assessing eyelid closure, yawning, performing hair styling and makeup, observing drivers’ posture/reaching behind within the vehicle, and operating the radio or dashboard controls. However, these subtopics were not the primary focus of the present study, as they do not pertain directly to smartphone-related distractions during driving.

**Figure 4:**
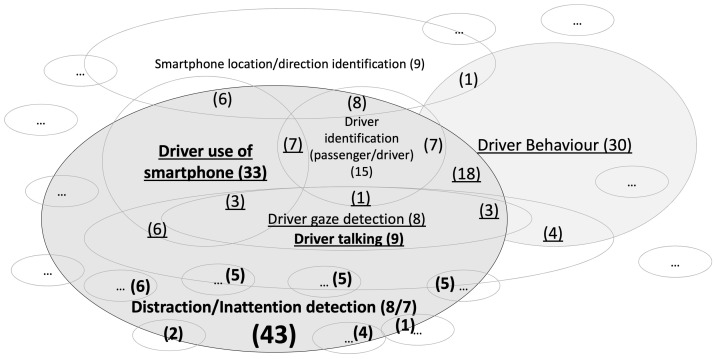
Topics prevailing, their relation, and number of papers (in round brackets) referring to those topics.

The majority of the papers, as shown in Figure 4, address one of the following main topics, driver distraction, driver behaviour, driver use of smartphones, and driver identification. However, several reviewed papers may also address more than one of these topics in combination:

(a) *Driver distraction* includes research dealing with driver state monitoring. Although the focus of the paper was to review scientific work on driver distraction monitoring, we decided to also include work on two related topics, driver drowsiness (e.g., [54]) and driver inattention detection (e.g., [104]), in our review and to group them under driver state monitoring whenever smartphone sensors are used. In addition, we chose to include these papers because parts of the applied methodology, such as detecting eye gaze (e.g., [49,94,116]), face direction (e.g., [116]), head poses (e.g., [53]), or calculating eye closure (e.g., [49]) or yawning status (e.g., [52]), can be applied to distracted driving monitoring, too.

(b) *Driver behaviour* is a rather complex concept that is considered to include the characterisation of human driving behaviour to fulfil a specific purpose, such as aggressive or risky driving, unsafe or distracted or low-responsiveness driving (cf. driver distraction topic above), and energy-efficient or eco-friendly driving style. Specifically, *distracted driving behaviour* includes talking to people in the vehicle, eating and drinking, talking or texting on a smartphone, adjusting vehicle controls such as navigation or infotainment, and looking away from the road; while many authors focus explicitly on approaches to detect driver distraction (e.g., [49,54,69,116]), others may focus on detecting driving behaviour in a more general way (e.g., [89]. However, parts of the methodology used by these authors can also be applied in the context of distracted driver monitoring. Other authors are developing technical enablers for better detection and warning systems that capture vehicle movements and manoeuvres [76,109,112] or detect harsh driving events [83,108].

(c) *Driver use of smartphones* includes observing the driver performing a secondary task while driving, using a smartphone. This may be reported in an unspecified way (i.e., smartphone use) or, more specifically, holding the smartphone, the smartphone is placed near the ear, or the smartphone is being held and operated (texting, calling, browsing, etc.). Papers identify interactions with the smartphone, in general [73], or talking on the phone [94]. Furthermore, parts of the approaches used can again be applied in distracted driver monitoring, too.

(d) *Driver identification (driver/passenger)* refers to identifying the person behind the wheel. This topic, along with the topic of identifying the location or direction of the smartphone (as a source of distraction), is included in this literature review, even though it is indirectly related to distracted driver monitoring (cf. studies such as smartphone location or direction [66], driver alerting [102], or distinguishing drivers from passengers [63,114]). When using smartphone sensors to detect distracted driving, it is important to differentiate between driver and passenger so as not to alert the wrong person.

### 4.3. Aggregated Results: Smartphone-Based Sensors and Detection Methods

To provide an overview of the papers and to answer RQ2, Table 5 explicitly shows which smartphone sensors were used per paper, using the categories camera, GNSS (e.g., GPS), IMU (e.g., accelerometer, gyroscope, magnetometer), microphone, or radio signals (e.g., WiFi).

The approaches described in the papers used either one, two, or three different types of smartphone data, e.g.:

- The smartphone camera (front and/or rear camera) to collect images or videos;
- Data from a GNSS (global navigation satellite system, often the US-developed Global Positioning System is used) to collect position data or to calculate vehicle speed;
- Data from the inertial measurement unit (IMU) of the smartphone, which usually includes an accelerometer, a gyroscope, and, in some cases, a magnetometer;
- Smartphone microphones to collect sound data;
- Different types of radio signals from the smartphone, e.g., WiFi signals or the radio signal connection between the smartphone and the base station/base transceivers.

**Table 5:** Literature result: 65 papers and the smartphone data type they use: camera (CAM), GNSS, IMU, microphone (MIC), or radio signals (RAD).

Author(s)	CAM	GNSS	IMU	MIC	RAD
Ahn et al., 2017 [65]			X		
Ahn et al., 2019 [66]			X		
Albert et al., 2016 [67]					
Alqudah et al., 2021 [68]			X		
Baheti et al., 2018 [69]	X				
Bergasa et al., 2014 [54]	X	X	X	X	
Berri et al., 2014 [71]	X				
Bo et al., 2013b [72]			X	X	
Bortnik and Lavrenovs, 2021 [73]					
Caird et al., 2014 [74]					
Castignani et al., 2015 [75]			X		
Chen et al., 2015 [76]			X	X	
Chu et al., 2014 [77]			X	X	
Chuang et al., 2014 [78]	X				
Dai et al., 2019 [79]				X	
Dua et al., 2019a [49]	X				
Dua et al., 2019b [49]	X				
Eraqi et al., 2019 [50]	X				
Gelmini et al., 2020 [46]		X	X		
He et al., 2014 [80]			X		
Hong et al., 2014 [81]		X	X		
Janveja et al., 2020 [52]	X				
Jiao et al., 2021 [82]	X				
Johnson et al., 2011 [83]	X	X	X		
Kapoor et al., 2020 [48]	X				
Kashevnik et al., 2021 [5]	X			X	
Khurana and Goel, 2020 [84]	X				
Koukoumidis et al., 2011 [85]	X	X	X		
Li et al., 2019 [86]		X	X		X
Lindqvist and Hong, 2011 [87]					
Liu et al., 2017 [88]	X	X	X		
Ma et al., 2017 [89]		X	X	X	
Mantouka et al., 2019 [90]		X	X		
Mantouka et al., 2022 [91]		X	X		
Meiring et al., 2015 [92]					
Meng et al., 2015 [93]	X	X			
Mihai et al., 2015 [53]	X				
Nambi et al., 2018 [94]	X				
Omerustaoglu et al., 2020 [51]		X	X		
Othman et al., 2022 [95]	X	X	X		
Pargal et al., 2022 [96]				X	
Park et al., 2018 [97]			X		
Paruchuri and Kumar, 2015 [63]	X				
Punay et al., 2018 [98]	X				
Qi et al., 2019a [99]		X	X	X	
Qi et al., 2019b [100]	X	X	X	X	
Rachmadi et al., 2021 [101]			X		
Shabeer and Wahidabanu, 2012 [62]					X
Singh et al., 2014 [102]	X				
Song et al., 2016 [64]				X	
Torres et al., 2019 [103]		X	X		
Tortora et al., 2023 [104]	X	X	X		
Tselentis et al., 2021 [105]		X	X		
Wang et al., 2016 [106]			X		
Vasey et al., 2018 [107]		X	X		
Vlahogianni and Barmpounakis, 2017 [108]		X	X		
Woo and Kulic, 2016 [109]		X	X		
Xiao and Feng, 2016 [111]	X				
Xie and Zhu, 2019 [110]		X	X		
Xie et al., 2018 [112]		X	X		
Xie et al., 2019 [113]		X	X		
Yang et al., 2012 [114]				X	
Yaswanth et al., 2021 [115]			X		X
You at al., 2013 [116]	X	X	X		
Ziakopoulos et al., 2023 [117]	X	X	X		
Total	27	26	37	12	3

In many cases, a camera that is recording the driver while driving is placed inside the car, and the taken images are used to classify the driver’s actions.

Computer-vision-related research includes driver gaze detection or driver eye detection [49], driver eye closure detection, driver yawn detection [49], and driver pose detection [95], as well as other driver activity detection, such as attending their hair and makeup, reaching behind, operating the radio, eating, and drinking [50]. Audio-related research includes identifying driver talking activity and phone use [64,79].

There is much less work that combines data from multiple modalities for distracted driver detection tasks.

### 4.4. Aggregated Results: Summary of Tangible Results

The table below provides a structured overview of the reviewed papers and their results with respect to the objective of the SLR (answering RQ3). The objectives of the papers are reported in terms of the use of smartphones, while the analysis methods and accuracy results are also listed when reported by the paper authors.

The research objectives of the reviewed papers range from the detection of when a person enters the vehicle [65], the classification of users into drivers and passengers [63,66,77,114], the identification of the driver’s interaction with the smartphone [64,73,84,106], the classification of driving events and driving styles [68,75,83,90], the detection of distracted drivers [48,49,50,51,52,69,113], the detection of inattentive drivers [49,53,54,111], the detection of gaze direction for distracted driving [78], to the identification of the driver’s speech direction [79].

The analysis methods used include machine learning models [66,68,77,78,79,84,86,90,103,105,107,109,110,111,112], neural networks [48,49,50,51,69,99,100], computer vision [53,71,85,93,116], fuzzy logic [65,75], signal processing [76,80,83,88,89,106], and smartphone app design [5,94], to name a few examples of popular approaches.

The results obtained vary, largely depending on the scope of the research and the datasets used, and include, for example, the identification of the smartphone with about 90% accuracy [66], the classification of driving events with over 90% accuracy [75], or even with over 98% accuracy [68], the detection of distracted driving on images with an accuracy of 90% [50], with 96% in [69] or even with close to 100% [48].

**Table 6:** Literature result (Part 1 of 5): summary table of papers reporting on objective, analysis methods, and obtained results.

Author(s)	Objective	Analysis Methods	Results
Ahn et al., 2017 [65]	Detect when a person is about to enter a vehicle by analysing the movement trajectory of the smartphone	Fuzzy Inference System, electromagnetic field (EMF) fluctuations	91.1% to 94.0% accuracy; maintains at least 87.8% accuracy regardless of smartphone position and vehicle type
Ahn et al., 2019 [66]	Classify users into drivers and passengers and whether they have entered a vehicle	Bayesian classifier	Identifies the driver’s smartphone with 89.1% average accuracy
Albert et al., 2016 [67]	Identify smartphone apps that have the greatest potential to reduce risky driving behaviour	Apps mapping, Analytic Hierarchy Process (APH)	Texting prevention and Green Box are unlikely to be accepted and used; collision warning and voice control are expected to gain public support
Alqudah et al., 2021 [68]	Classify driving events such as high speed, low speed, stop, and U-turn using smartphone sensors	SVM, decision trees, Discriminate Analysis, Naïve Bayes, KNN, ensembles	Classify events with over 98% accuracy using decision trees
Baheti et al., 2018 [69]	Detect distracted drivers and the type of distraction, such as texting, talking on a mobile phone, eating, or drinking	CNN (VGG-16 architecture)	94.44% accuracy on test set; adding dropout, L2 weight regularisation, and batch normalisation increases accuracy to 96.31% on test set
Bergasa et al., 2014 [54]	Detect inattentive driving and provide feedback to the driver, assessing their driving and warning them if their behaviour is unsafe	Drowsiness score uses lane drifting and lane weaving signals to infer drowsiness; distraction score based on sudden longitudinal and lateral movements	Data from 12 drivers in two different studies; detects some inattentive driving behaviours and achieves an overall accuracy of 82% with a recall of 92%
Berri et al., 2014 [71]	Present an algorithm for extracting features from images to detect the use of mobile phones by drivers	Computer vision and machine learning (SVM for classification)	Average accuracy of 91.57% for the set of images analysed
Bo et al., 2013b [72]	Detect drivers and passengers, and whether a smartphone is being used for texting	Classification with hidden Markov model (HMM)	Classification accuracy of 87% and precision of 96.67% across 20 different driving and parking cases
Bortnik and Lavrenovs, 2021 [73]	Identify the driver’s interaction with the smartphone, such as app activity, call activity, or screen activity	Android dumpsys diagnostic data	N/A
Caird et al., 2014 [74]	Presents a meta-study on texting and driving	N/A	N/A
Castignani et al., 2015 [75]	Detect events related to driving style and scores drivers	Fuzzy logic, principal component analysis (PCA)	The developed system shows more than 90% accuracy in detecting events in an experiment with 10 drivers along a predefined route
Chen et al., 2015 [76]	Detect and differentiate between different vehicle steering patterns, such as lane changes, turns, and driving on winding roads	Signal processing, Kalman filter	High detection accuracy: 100% for right and left turns, 93% for lane changes, 97% for curvy roads
Chu et al., 2014 [77]	Detect whether a smartphone user in a vehicle is the driver or a passenger	Machine learning approach	Early prototypes on Android and iOS show over 85% accuracy with 6 users in 2 different cars
Chuang et al., 2014 [78]	Estimate driver gaze direction to detect driver distraction	Multi-class linear support vector machine (SVM) classifier	Classification accuracy between 86.4% and 97.4%.
Dai et al., 2019 [79]	Identification of the driver’s direction of speech (namely, front, right, and rear)	K-means clustering algorithm	95% accuracy on average for different phone placements, at least 92.2% accuracy for three scenarios, 90.3% accuracy when the window is open in the presence of outside noise

**Table 7:** Literature result (continued, Part 2 of 5): summary table of papers reporting on objective, analysis methods, and obtained results.

Author(s)	Objective	Analysis Methods	Results
Dua et al., 2019a [49]	Detect and assess driver attention using the front camera of a windscreen-mounted smartphone	Neuronal networks, CNNs, and GRUs	The driver’s attention rating had an overall agreement of 0.87 with the ratings of 5 human annotators
Dua et al., 2019b [49]	Identify driver distraction based on facial characteristics (head position, eye gaze, eye closure, and yawning)	CNN (generic features) and GRU or (CNN + GRU)	The automatically generated rating has an overall agreement of 88% with the ratings provided by 5 human annotators; the attention-based model outperforms the AUTORATE model by 10% accuracy on the extended dataset
Eraqi et al., 2019 [50]	Detect 10 different types of driver distraction (including talking to passengers, phone calls, and texting)	Deep learning; ensemble of convolutional neural networks	New public dataset, detection with 90% accuracy
Gelmini et al., 2020 [46]	Driving style risk assessment based on speeding, longitudinal acceleration, lateral acceleration, and smartphone use while driving	Thresholds used for profiling drivers and detecting smartphone usage	Median phone usage, no accuracy indicators used
He et al., 2014 [80]	Present a seat-level location of smartphones in a vehicle to identify who is sitting where	Signal processing: reference frame transformation, event detection, left/right identification, front/back identification	Position accuracy between 70% and 90% (best case)
Hong et al., 2014 [81]	Detect a person’s driving behaviour via an Android-based in-vehicle sensor platform	Machine learning approach (Naïve Bayes classifier)	Average model accuracy with all three sensors was 90.5%, and 66.7% with the smartphone only
Janveja et al., 2020 [52]	Introduce a smartphone-based system to detect driver fatigue and distraction (mirror scanning behaviour) in low-light conditions	For distraction detection, statistics are calculated if the driver is scanning their mirrors at least once every 10 s continuously during the drive	NIR LED setup: 93.8% accuracy in detecting driver distraction
Jiao et al., 2021 [82]	Recognise actions of distracted drivers	Hybrid deep learning model, OpenPose, K-means, LSTM	Accuracy depending on processing step (up to 92%)
Johnson et al., 2011 [83]	Detect and classify driving events, such as left/right manoeuvres, turns, lane changes, device removal, and excessive speed and braking	Manoeuvre classification with the DTW algorithm	U-turn correctly identified 23% of the time (using accelerometer), 46% of the time (using gyroscope), 77% of the time (combined sensors), 97% of aggressive events correctly identified
Kapoor et al., 2020 [48]	Provide a real-time driver distraction detection system that detects distracting tasks in driver images	Convolutional neural networks (CNNs)	Accuracy for 4 classes (e.g., calling or texting on a cell phone) reaches 98–100% when fine-tuned with datasets such as the State Farm Distracted Driver Dataset
Kashevnik et al., 2021 [5]	Provide an audio-visual speech recognition corpus for use in speech recognition for driver monitoring systems	Corpus creation, development of smartphone app	Corpus (audio-visual speech database with list of phrases in Russian language, 20 participants)
Khurana and Goel, 2020 [84]	Detect smartphone use by drivers using in-device cameras	Random forest classifiers (machine learning models) for 2 scenarios: a) docked, b) in-hand	Approximately 90% accuracy in distinguishing between driver and passenger. Cannot collect data for phones in handheld position
Koukoumidis et al., 2011 [85]	Detect traffic lights using the smartphone camera and predict their timing	Machine learning (Support Vector Regression)	Accuracy of traffic signal detection (87.6% and 92.2%) and schedule prediction (0.66 s, for pre-timed traffic signals; 2.45 s for traffic-adaptive traffic signals)

**Table 8:** Literature result (continued, Part 3 of 5): summary table of papers reporting on objective, analysis methods, and obtained results.

Author(s)	Objective	Analysis Methods	Results
Li et al., 2019 [86]	Introduce the WisDriver system, which detects 15 different dangerous driving behaviours	Multiple approaches for signal processing (sliding window, mean absolute deviation): PCA, DTW, discrete wavelet transform (DWT)	CSI plus sensor can achieve up to 92% detection accuracy
Lindqvist and Hong, 2011 [87]	Conduct user interaction research to design driver-friendly smartphone applications that do not distract the driver	Interaction designs (no analysis)	Initial interaction designs for Android apps
Liu et al., 2017 [88]	Recognition of internal driver inputs (e.g., steering wheel angle, vehicle speed, and acceleration) and external perceptions of the road environment (e.g., road conditions and front view video)	Signal processing, filtering approaches, deep neural networks	Estimate steering wheel angle with an average error of 0.69, infer vehicle speed with an error of 0.65 km/h, and estimate binary road conditions with 95% accuracy
Ma et al., 2017 [89]	Propose a scheme to identify three dangerous driving behaviours, speeding, irregular change in direction and abnormal speed control	Coordinate reorientation, sensor error estimation, data correction, speed estimation, turn-signal identification	Kalman filter approach: average precision and recall for direction change and abnormal speed detection are 93.95% and 90.54%, respectively,
Mantouka et al., 2022 [91]	Identify unsafe driving styles and provide personalised driving recommendations	Two-stage K-means clustering	Summary statistics on collected trip data
Mantouka et al., 2019 [90]	Identify driver safety profiles from smartphone data and distinguish normal driving from unsafe driving	Unsupervised learning: two-stage K-means clustering approach	7.5% of the trips are characterised by distracted driving
Meiring et al., 2015 [92]	Review solutions and approaches to driving style analysis to identify relevant ML and AI algorithms	N/A	N/A
Meng et al., 2015 [93]	Develop a system that extends the driver’s view in all directions by using cameras from multiple cooperating smartphones in surrounding vehicles	Image processing	System detects a vehicle within 111 ± 60 ms
Mihai et al., 2015 [53]	Develop a system to determine the orientation of the driver’s head to infer visual attention	Image processing (OpenCV)	Feasibility tests in two scenarios, no numbers given
Nambi et al., 2018 [94]	Develop a windscreen-mounted, smartphone-based system to monitor driving behaviour (including driver states)	Android app: uses OpenCV, TensorFlow, and custom libraries (DNN and SVM)	Demonstration case, no further information provided by the authors
Omerustaoglu et al., 2020 [51]	Introduce a two-stage driver distraction detection system that integrates vehicle sensor data into a vision-based distraction detection model	CNN, LSTM-RNN on sensor and image data together; model tuning and transfer learning (from StateFarm to own dataset)	Increased overall accuracy to 85% compared to using only image data. Increased driver detection accuracy to 85% using sensor data.
Othman et al., 2022 [95]	Introduction of a driver state identification dataset synchronised with vehicle telemetry data	Dataset provision, unsupervised learning approach (K-means)	Clustered, labelled dataset
Pargal et al., 2022 [96]	Present an approach to detecting whether a smartphone is being used by the driver	Spectral analysis, power analysis of noise features, acoustic-based smartphone localisation	F1 scores from 0.75 to 0.875 for different smartphone placement scenarios

**Table 9:** Literature result (continued, Part 4 of 5): summary table of papers reporting on objective, analysis methods, and obtained results.

Author(s)	Objective	Analysis Methods	Results
Park et al., 2018 [97]	Detect the location and direction of the driver’s phone, as well as in-car activities, such as walking towards the vehicle, standing near the vehicle while opening a door, and starting the engine	Electromagnetic field (EMF) fluctuations are analysed	The driver’s phone was identified with 83–93% true positive rate and achieved 90–91% true negative rate
Paruchuri and Kumar, 2015 [63]	Detects smartphone location and distinguishes drivers from passengers	Image comparison (angle difference) with reference images for the localisation of the smartphone (driver’s seat vs. passenger seats), based on the distance between images	15 out of 38 images were registered incorrectly
Punay et al., 2018 [98]	Focus on a safer driving experience by providing an Android application for non-distracted driving	Thresholds are used, i.e., the system detects if the speed is higher than a certain threshold	N/A
		Prototype only	
Qi et al., 2019a [99]	Detect in-car human activity, such as chatting, and contextual information (clear vs. crowded) based on vehicle dynamics (braking and turning)	Convolutional neural network (CNN) for the audio	Average accuracy of 90% across 7 different activities
Qi et al., 2019b [100]	Classify driving events, such as turning, braking, and lane changes, using sensor data, while cameras and microphones are used to identify objects in front view and blind spots and estimate head position	Deep learning inference (Nvidia TensorRT)	Average of 90% event detection accuracy
Rachmadi et al., 2021 [101]	Present a driver abnormal behaviour classification system	Enhanced multi-layer perceptron (MLP)	97,5% accuracy and 45 ms processing time
Shabeer and Wahidabanu, 2012 [62]	Detect driver phone calls	Threshold value cutoff of the receiving RF signal	N/A
Singh et al., 2014 [102]	Blind spot vehicle detection	Two approaches: intensity variation and contour matching	Detect and alert the driver with 87% accuracy
Song et al., 2016 [64]	Detect driver phone calls	Similarity based on threshold: voice feature model	TPR is over 98% for 3 different evaluated passenger positions, over 90% with noise impact, 80% when three people are talking, and 67% when 4 people are talking
Torres et al., 2019 [103]	Use data from smartphone sensors to distinguish between driver and passenger when reading a message in a vehicle	Machine learning (various models): three eager learners (SVM: DT, LR), three ensemble learners (RF, ADM, GBM), and one deep learning model (CNN)	Performance values accuracy, precision, recall, F1, and Kappa: CNN and GB models had the best performance
Tortora et al., 2023 [104]	Develop Android application to detect distracted driving behaviour	Distraction score based on different distraction activities and detection methods	Application presentation (no KPIs)
Tselentis et al., 2021 [105]	Driving behaviour analysis using smartphone sensors to provide driver safety scores and driver clustering	K-means driver clustering (based on event compute in a trip such as phone use, speeding, harsh braking, etc.)	Descriptive statistics, definition of driver characteristics for each cluster (moderate, unstable, cautious drivers)
Wang et al., 2016 [106]	Present an approach based on smartphone sensing of vehicle dynamics to determine driver phone use	Signal processing: compute centripetal acceleration using smartphone sensors and compare to those measured by a simple plug-in reference module	Approach achieves close to 90% accuracy with only a few with less than 3% FPR
Vasey et al., 2018 [107]	Driver emotional arousal detection	Machine learning classifier (decision tree, SVM, NN)	N/A, concept only

**Table 10:** Literature result (continued, Part 5 of 5): summary table of papers reporting on objective, analysis methods, and obtained results.

Author(s)	Objective	Analysis Methods	Results
Vlahogianni and Barmpounakis, 2017 [108]	Detect driving events such as braking, acceleration, left and right cornering	Rough set theory and own classifier (MODLEM), compared to MLP, C4.5 decision trees, and ZeroR	Smartphone accuracy is 99.4% and OBD-II device accuracy is 99.3%; TPRs are 88% and 86% and FPRs are 0.3% and 0.4% for smartphone and OBD-II device, respectively,
Woo and Kulic, 2016 [109]	Propose a classifier-based approach for driving manoeuvre recognition from mobile phone data	SVM classifier, PCA	Average precision of 0.8158 and average recall of 82%. Balanced accuracy of 88%.
Xiao and Feng, 2016 [111]	Driver attention detection with 2 modules: a) gaze detection and b) road motion objects detection	Linear SVM classifier (module a); Lucas–Kanade optical flow with dynamic background compensation (module b)	93% accuracy for gaze estimation and 91.7% overall accuracy
Xie and Zhu, 2019 [110]	Manoeuvre-based driving behaviour (lane changing or turning) and classification amongst three labels (normal, drowsy, and aggressive)	ReliefF, random forest	Average F1 score of 70.47% using leave-one-driver-out validation
Xie et al., 2018 [112]	Classification of driving manoeuvres (i.e., braking, turning, stopping, accelerating, decelerating, lane changing) based on different feature extraction methods	Random forest classifier	F1 scores of 68%, 80%, and 87% on three different datasets
Xie et al., 2019 [113]	Driver distraction detection	Ensemble method of 4 classifiers: K-NN, Logistic Regression, Gaussian Naive Bayes, random forest	87% accuracy in distraction detection
Yang et al., 2012 [114]	Distinguish between passengers and drivers using smartphones by classifying the position of the smartphone	Threshold-based classification	Accuracy with calibrated thresholds: detection rate is over 90% and accuracy is around 95%
Yaswanth et al., 2021 [115]	Smartphone detection (classifier) and drivers’ action detection	N/A	N/A
You et al., 2013 [116]	Detect if drivers are tired or distracted (drowsy driving, inattentive driving) and identify various driving conditions such as tailgating, lane weaving, or drifting	Computer vision and machine learning (decision trees and SVM)	Precision and recall for face direction events: precisions are 68% for facing left, 79% for facing right, and 92% for eye state classification
Ziakopoulos et al., 2023 [117]	Investigate influence factors for driver distraction through smartphone use	230-driver experiment using the developed driving recording application and feedback questionnaire, XGBoost for distraction investigation	Deducted influence factors for driver phone use

**Table 11:** List of journals that published the papers.

List of Journals	No. of Papers
Sensors (Switzerland)	7
Accident Analysis and Prevention	3
IEEE Transactions on Biometrics, Behaviour, and Identity Science	3
International Journal of Interactive Mobile Technologies	2
Transportation Research Part C: Emerging Technologies	2
Advances in Intelligent Systems and Computing	1
Applied Soft Computing Journal	1
Data	1
IEEE Access	1
IEEE Intelligent Transportation Systems Magazine	1
IEEE Sensors Journal	1
IEEE Transactions on Intelligent Transportation Systems	1
IEEE Transactions on Mobile Computing	1
Journal of Advanced Transportation	1
Lecture Notes in Electrical Engineering	1
Lecture Notes of the Institute for Computer Sciences, Social-Informatics	1
and Telecommunications Engineering	
Mobile Information Systems	1
Procedia Engineering	1
Proceedings of the ACM on Interactive, Mobile, Wearable and	1
Ubiquitous Technologies	
Safety Science	1
Transport Policy	1
Total (Percentage)	33 (51%)

**Table 12:** List of conference venues where the papers were presented.

List of Conferences	No. of Papers
International Conference on Mobile Systems, Applications and Services	4
Conference on Human Factors in Computing Systems	2
IEEE Intelligent Vehicles Symposium	2
International Conference on Computing, Networking and Communications	2
International Conference on Mobile Computing and Networking	2
IEEE Computer Society Conference on Computer Vision	1
and Pattern Recognition Workshops	
IEEE Conference on Intelligent Transportation Systems	1
IEEE International Conference on Automatic Face and Gesture Recognition	1
IEEE International Conference on Computer Communications	1
IEEE International Conference on Mobile Ad Hoc and Smart Systems	1
IEEE International Conference on Systems, Man, and Cybernetics	1
IEEE Pacific Rim Conference on Communications, Computers	1
and Signal Processing	
IEEE Vehicular Networking Conference	1
International ACM Conference on Automotive User Interfaces	1
and Interactive Vehicular Applications	
International Conference on Advanced Information Networking	1
and Applications	
International Conference on Communication Systems and Networks	1
International Conference on Computer Vision Theory and Applications	1
International Conference on Information, Intelligence, Systems	1
and Applications	
International Conference on Intelligent Transport Systems	1
International Conference on Neural Computation, Fuzzy Systems	1
and Knowledge Discovery	
International Conference on Mobile Data Management	1
International Conference on Orange Technologies	1
International Conference on Transportation Information and Safety	1
International Electronics Symposium	1
Workshop on Mobile Computing Systems and Applications	1
Total (percentage)	32 (49%)

## 5. Discussion

In our study, based on a systematic literature review, we evaluated the current state of the art in smartphone-based driver behaviour monitoring and driver distraction detection according to four criteria: the objectives of the approaches, the types of approaches, the sensors used, and the results obtained.

Before discussing the contributions of our study, we would like to point out our limitations, including the paper selection bias. We extended a preliminary literature review published in a conference paper and built our results on it. In addition, we selected three scholarly databases, IEEE Xplore, Scopus, and Web of Science, to search for recent literature on the research topic. To supplement our use of these three databases and to include additional relevant literature that we might otherwise have missed, we also conducted backward and forward searches using Google Scholar through a rigorous snowballing research process that we performed; while we are confident that our final sample represents the current literature on our research topic, it is possible that we may have missed existing work, despite our rigorous review approach. In addition, we based our research on peer-reviewed scientific papers, excluding white papers, technical reports, or company press releases. As a result, we may have missed out on industry experiences.

Furthermore, our paper is a literature review, and within the reviewed scientific literature, the findings are derived from developed and evaluated research prototypes. There is no commercially available solution for a smartphone-based driver distraction detection system, and therefore no real-life experience of people using such a system could be integrated. From a practitioner’s perspective, we supplemented the results of our study with practical domain knowledge from our expertise in automotive engineering.

Driver behaviour monitoring is a broad area of research with a variety of methods and approaches, and distracted driver monitoring is an important subset of this research. In addition, while modern smartphones pose a risk of distracting drivers when used in cars, they also offer great potential for enabling solutions to detect driver inattention and distraction due to their built-in sensory and computational capabilities.

Against this background, this paper offers an extended systematic literature review to capture the state of the art in smartphone-based distracted driving monitoring approaches extending a previously carried out literature review [23]. Specifically, this paper sheds light on three research questions: What types of smartphone-based approaches have been published? What types of smartphone sensors and detection methods have been used? And what tangible results have been achieved?

Driver behaviour monitoring is a complex problem, and to date, there appears to be no commercially available smartphone application for distracted driving monitoring on the market. However, there are some presented research prototypes (e.g., [48,94]) and outlined individual approaches to explore the technical feasibility, which are reviewed in this paper. If these individual approaches were combined in systems to detect driver distraction and evaluated in real-world situations, they would certainly have great exploitation potential.

The results of our review suggest that a lot of research using smartphone sensors seems to be quite successful in achieving good machine learning KPIs, such as high recognition accuracies or good F1 scores, to name two examples. This raises the question of why we do not yet see smartphone-based distraction detection systems in action.

We suggest several reasons why such systems are not yet available on the market. Firstly, researchers typically work on methods and theories, and design system prototypes to test and evaluate the success of their models and methods. Secondly, they work on mostly available datasets for training machine learning models, or they collect their own, mostly limited datasets, which they use to train and apply their models. These research datasets tend to be very homogeneous, and when divided into 80% training and 20% validation sets, the machine learning models trained usually provide good accuracy, which also explains the high accuracies achieved in deriving the distracted driving task from images, ranging from 90% to almost 100%. Thirdly, if these models were evaluated in real conditions with distracted drivers in real vehicles, the input data for these models would be much more diverse; for example, for image data, people would behave differently, look differently, camera angles would be different, lighting conditions would be different, there would be sun reflections, and these models would also have to work during night driving. Therefore, and fourthly, the trained models are often tested in laboratory or lab-like conditions, and if in real life, in standardised conditions to allow good results to be accepted at conferences or in journal papers.

Traditional driver state monitoring systems in cars use the sensors in the steering wheel and detect driver inattention and distraction by examining steering behaviour, but relying on inferring driver behaviour from sensitive monitoring of steering movements and steering wheel reversal rates [118] can have several limitations and may not capture all phenomena with high enough accuracy. Therefore, researchers have explored a variety of further approaches to driver monitoring, using multiple cameras, infrared cameras, or in-cabin time of flight sensors for professional industry-proof systems. However, these systems were not the focus of our literature review as we wanted to shed light on the use of smartphones to detect driver distraction.

Due to the advent of partially automated driving, another push has been made to offer camera-based driver monitoring systems in a series of vehicles. For instance, the manufacturer Tesla has integrated in-cabin camera-based driver monitoring systems to prevent misuse of its autopilot feature (Tesla has activated its in-car camera to monitor drivers using Autopilot, https://techcrunch.com/2021/05/27/tesla-has-activated-its-in-car-camera-to-monitor-drivers-using-autopilot/ accessed on 24 July 2023). In addition, the updated European Vehicle General Safety Regulation (new rules to improve road safety and enable fully driverless vehicles in the EU, https://ec.europa.eu/commission/presscorner/detail/en/ip_22_4312 accessed on 24 July 2023) will require some form of alertness and drowsiness warning in newly registered European vehicles.

While the use of smartphones while driving continues to pose a serious risk to safety, numerous methods from different fields have been proposed and put into practice to lessen their adverse impacts. These include laws prohibiting the use of handheld mobile devices, bans on texting, strict enforcement of the laws, establishing designated texting zones on highways, educational campaigns, and suggestions to automobile manufacturers to restrict communication through electronic devices integrated into their vehicles, such as browsing, entertainment, and texting. Nevertheless, there is an ongoing debate about the practicality, efficacy, and acceptability of these measures.

From the studies reviewed, depending on the specific sensor combinations used (see Table 5), the smartphone can be placed in different locations to collect data, e.g, (a) for gaze sensing, it needs to be placed on the dashboard or mounted on the windshield facing the driver; (b) for GNSS sensing, it needs to be placed on the dashboard or near the windshield to ensure clear sky visibility for accurate GPS-based position and speed data; (c) for IMU sensing, it needs to be placed in a stable position in the vehicle, such as on the centre console or mounted on the driver’s seat, to record precise motion and orientation changes; and (d) for microphone sensing, it needs to be placed in the cabin, preferably on the dashboard or near the centre console, to record auditory cues and driver interactions. Details of such placements are typically discussed in the papers whose studies were examined in our review. Depending on the sensor combinations used, the smartphone may need to be mounted in a central location within the vehicle, such as on the dashboard or windshield, and specialised smartphone holders or mounts designed for in-vehicle use are typically used. However, there are approaches that allow the use of IMU sensors without the need to mount the smartphone in a fixed position. These align the IMU sensor’s coordinate reference system with the vehicle’s coordinate reference system.

## 6. Conclusions and Outlook

Our paper provides an extended systematic literature review of smartphone-based distracted driving monitoring approaches [23]. This extended review includes a total of 65 relevant scientific papers that address smartphone-based distraction detection approaches, the smartphone sensors and detection methods used, and the results obtained. We analysed all papers in terms of their objectives, the analytical methods used (including ML and AI), and the results obtained in terms of KPIs, where these were provided. In doing so, we are contributing to the growing literature on driver monitoring and distraction detection systems.

The core literature in this field encompasses papers focusing on driver distraction, driver inattention, and driver use of smartphones (with a specific emphasis on calling or texting), as well as papers addressing issues like identifying the driver from the passenger and determining smartphone location/direction. Additionally, studies analysing driver behaviour associated with risky driving are also relevant to the topic at hand. A variety of other subtopics related to driver distraction were investigated in the literature and were discussed, too.

Based on the results of this literature review, our paper calls for further contributions on smartphone-based driver monitoring systems, integrating the different approaches presented by the authors, as well as integrating all available sensors on smartphones to enable a benchmark against industry-grade in-vehicle driver monitoring systems. Moreover, our work calls for more applications in real life, as they are currently missing. We have identified a gap in research carried out in real-world conditions that is systems performing reliably across diverse driving scenarios, weather conditions, and individual driving styles [119]. This is a significant challenge, which necessitates extensive testing and validation. In order to contribute to this future direction, there are several technical limitations as this requires advanced technologies, such as computer vision, machine learning, and sensor fusion, to be combined in effective smartphone-based driver distraction detection systems. Effective (i.e., highly accurate and reliable) systems in real-time scenarios are challenging, especially given the diverse and complex driving environments vehicles are found in. Another wide future avenue for research is sensor appropriateness, accuracy, and consistency. Choosing the most appropriate sensors (either smartphone-based or not sensors) that can provide increased effectiveness is crucial and complex as sensors vary in quality, calibration needs, and performance. The placement of the sensors also leads to measurement inconsistencies and inaccuracies that can lead to false positives or negatives in distraction detection, which may jeopardise the system’s credibility.

While smartphone-based driver monitoring systems would allow retrofitting of vehicles with no or limited driver monitoring systems and would likely extend the life cycle of vehicles, the authors expect that available in-vehicle sensors will soon surpass smartphone sensors and cameras. However, new hardware that can connect to smartphones or act as a standalone system, such as AI cameras, will provide novel opportunities for “bring your own device” driver monitoring systems in the future. This introduces concerns about user data privacy and security, which is another future direction for research, as using personal device data for driver monitoring could raise legal and ethical issues related to consent, data ownership, and the potential misuse of sensitive information. Moreover, regulatory approvals and standardisation processes can be significant barriers to the development of these systems, in addition to cultural and driver behaviour changes, which are complex to achieve, even with the technology in place. Addressing the root causes of driver distraction and promoting safer habits may require more complex and multi-faceted approaches to occur than just overcoming the technical challenges.

## Figures and Tables

**Table 1:** Existing literature reviews: summary table of literature reviews on driver distraction detection.

Author(s)	Scope	Limitations
Dong et al., 2010 [3]	Focus on driver inattention monitoring	No in-depth review on smartphone-based systems
Kashevnik et al., 2021 [32]	Present holistic framework for detecting driver distraction	No detailed review on smartphone aspects
Lee et al., 2008 [6]	Define driver distraction	No focus on smartphone aspects
Oviedo-Trespalacios, O., 2016 [24]	Focus on aspects of distraction coming from the use of mobile phones inside a car	Does not consider smartphone as a tool or data source for approaches to prevent driver distraction
Young et al., 2007 [2]	Concentrate on distractions coming from inside a vehicle	No consideration of smartphones as tools or data source for approaches to prevent driver distraction

**Table 2:** Scoping and selected paper results.

Database	Scoping Step	Selecting Step
IEEE	14	3
Scopus	16	7
Web of Science	48	10
In total	78 (60 unique)	20 (16 unique)

**Table 3:** Statistics of the selection steps of papers.

Researcher	Initial Analysis	Iteration 1	Iteration 2	Iteration 3
1	Incl.: 17 Excl.: 52 Maybe: 9	Incl.: 20 Excl.: 58	Incl.: 18 Excl.: 60	Incl.: 16 Excl.: 62
2	Incl.: 11 Excl.: 65 Maybe: 2	Incl.: 11 Excl.: 67	Incl.: 18 Excl.: 60	Incl.: 16 Excl.: 62

## Data Availability

All data generated are included in this study.
